# Cancer-associated *ASXL1* mutations may act as gain-of-function mutations of the ASXL1–BAP1 complex

**DOI:** 10.1038/ncomms8307

**Published:** 2015-06-22

**Authors:** Anand Balasubramani, Antti Larjo, Jed A. Bassein, Xing Chang, Ryan B. Hastie, Susan M. Togher, Harri Lähdesmäki, Anjana Rao

**Affiliations:** 1 Department of Signaling and Gene Expression, La Jolla Institute for Allergy and Immunology, 9420 Athena Circle, La Jolla, California 92037, USA; 2 Sanford Consortium for Regenerative Medicine, La Jolla, California 92037, USA; 3 Department of Information and Computer Science, Aalto University School of Science, Aalto FI-00076, Finland; 4 Department of Pharmacology, University of California, San Diego, California 92093, USA; 5 Moores Cancer Center, University of California, San Diego, California 92093, USA

## Abstract

ASXL1 is the obligate regulatory subunit of a deubiquitinase complex whose catalytic subunit is BAP1. Heterozygous mutations of *ASXL1* that result in premature truncations are frequent in myeloid leukemias and Bohring–Opitz syndrome. Here we demonstrate that ASXL1 truncations confer enhanced activity on the ASXL1–BAP1 complex. Stable expression of truncated, hyperactive ASXL1–BAP1 complexes in a haematopoietic precursor cell line results in global erasure of H2AK119Ub, striking depletion of H3K27me3, selective upregulation of a subset of genes whose promoters are marked by both H2AK119Ub and H3K4me3, and spontaneous differentiation to the mast cell lineage. These outcomes require the catalytic activity of BAP1, indicating that they are downstream consequences of H2AK119Ub erasure. In bone marrow precursors, expression of truncated ASXL1–BAP1 complex cooperates with TET2 loss-of-function to increase differentiation to the myeloid lineage *in vivo*. Our data raise the possibility that ASXL1 truncation mutations confer gain-of-function on the ASXL–BAP1 complex.

## Abstract (web summary)

Premature truncations of ASXL1, a subunit of a deubiquitinase complex, are frequent in myeloid leukemia. Here, the authors show that expression of truncated ASXL1 in a haematopoietic precursor cell line enhances the deubiquitinase activity and leads to differentiation to the mast cell lineage.

## Graphical abstract

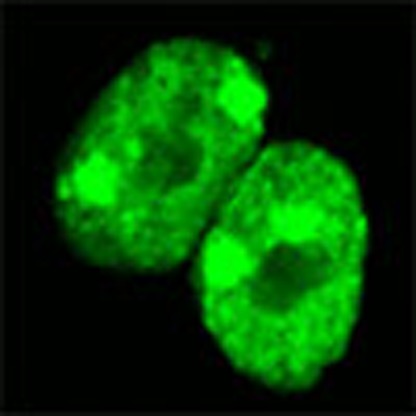

Monoubiquitination of lysine 119 on histone H2A (H2AK119Ub) is a histone modification associated with a repressed chromatin state. H2AK119Ub is deposited and removed by protein complexes containing polycomb group proteins^1^,^2^. Polycomb repressive complex 1 (PRC1) contains the E3 ligases RING1A/RING1B in humans and monoubiquitinates H2A at lysine 119 (H2AK119)^2^. PRC2 contains the histone 3 lysine 27 (H3K27) methyltransferase EZH2 and its partners EED and SUZ12, and produces the H3K27 trimethyl (H3K27me3) mark^3^,^4^,^5^,^6^. H2AK119Ub and H3K27me3 play synergistic roles in the establishment and maintenance of Polycomb-mediated gene repression^7^,^8^.

The precise sequence of events that govern deposition of the H2AK119Ub and H3K27me3 marks in mammalian cells is of considerable interest. Based on the initial observation that CBX family proteins containing H3K27me3-specific chromodomains co-purified with RING1B, RING1A and PCGF4 (polycomb group RING finger protein 4) in HeLa cells^9^, it was proposed that H2AK119Ub was deposited exclusively by the PRC1 complex, in a manner that involved recruitment of CBX family proteins to H3K27me3-marked regions of chromatin, followed by H2A ubiquitylation facilitated by RING1A/RING1B. This notion prevailed for over a decade. However, subsequent studies have documented recruitment of RING1A and RING1B, and ubiquitylation of H2A at target sites in a PRC2-independent manner^10^,^11^, indicating that this model was at least partially incorrect.

How do PRC1 and PRC2 complexes recognize genomic regions they target, and is there a hierarchy that controls recruitment of these complexes? Three recent studies have provided considerable new insight into this question. Reinberg and colleagues^12^ discovered that PRC1 complexes containing RING1A and/or RING1B in mammalian cells could be subdivided into two classes depending on their subunit composition. All PRC1 complexes contain PCGFs, obligate cofactors of RING1A and RING1B that are homologues of *Drosophila* Posterior sex combs^9^. The six PCGF proteins act independently to form distinct PRC1 complexes that have been named PRC1.1 to PRC1.6 based on the PCGF family member that constitutes the complex^12^. ‘Canonical' PRC1 complexes contain RING1A, RING1B, CBX proteins and either PCGF2 or PCGF4, whereas ‘variant' PRC1 complexes contain RING1A and/or RING1B but lack CBX proteins, instead containing PCGF1, 3, 5 or 6 and either RYBP (RING and Yin Yang 1-binding protein) or YAF2 (Yin Yang 1-associated factor 2)^12^. The lack of an H3K27me3 recognition module in variant PRC1 complexes suggested that they are recruited in an H3K27me3- and PRC2-independent manner^12^. Klose and colleagues^13^ extended these studies by demonstrating that the variant PRC1 complexes PRC1.1, 1.3 and 1.5 can in fact deposit H2AK119Ub in a PRC2-independent manner. More importantly, they also established that H2AK119Ub deposited by the variant PRC1 complexes could recruit components of the PRC2 complex and promote deposition of H3K27me3 marks^13^. Independently, Jürg Müller and colleagues^14^ demonstrated that H2AK119Ub-containing oligonucleosomes can physically interact with components of the PRC2 complex *in vitro*. Collectively, these studies have put forth the idea that at some genomic regions, H2AK119Ub may play an essential role in promoting recruitment of the PRC2 complex that deposits H3K27me3.

Here we have employed the newly discovered ‘Polycomb repressive deubiquitinase complex' (PR-DUB) that deubiquitinates H2AK119Ub to examine the role of H2AK119Ub in Polycomb-mediated gene repression. The mammalian PR-DUB complex contains proteins of the ASXL (*Drosophila* additional sex combs (Asx-like)) family as essential partners required for the DUB activity of the catalytic subunit, BAP1 (BRCA1-associated protein 1)^15^. *ASXL1* mutations have been observed in a variety of haematological malignancies in humans^16^,^17^,^18^ and acute disruption of the *Bap1* gene in mice leads to development of myeloid cancers^19^. Most cancer-associated *ASXL1* mutations give rise to truncated proteins that retain the amino-terminal BAP1-interacting region of ASXL1 (ref. 15), but lose the carboxy-terminal plant-homeodomain (PHD) domain, and most often, three centrally located proline-rich regions (PPRs) as well^16^,^20^. Heterozygous *de novo* mutations of *ASXL1* (refs 21, 22) or *ASXL3* (ref. 23) that result in similar loss of C termini are thought to be the cause of at least some cases of Bohring–Opitz syndrome, a rare and fatal congenital disorder.

Here we identify leukemia-associated *ASXL1* mutations that aberrantly enhance the DUB activity of the ASXL1–BAP1 complex. We establish that stable ectopic expression of these hyperactive ASXL1–BAP1 complexes leads to depletion of ∼90% of total H2AK119Ub and reduction in bulk levels of H3K27me3 by ∼50%. By mapping the genome-wide distribution of H2AK119Ub and H3K27me3, we demonstrate that the two modifications overlap extensively, both at intergenic regions and in the vicinity of promoters: specifically, ∼74% of genomic regions marked by H2AK119Ub in EML haematopoietic cells also carried H3K27me3 marks. Further, we establish that the ability of the hyperactive ASXL1–BAP1 complex to deplete H3K27me3 is absolutely dependent on the catalytic activity of BAP1, indicating that H2AK119Ub plays an essential role in either recruiting or retaining the PRC2 complex at some genomic locations. Based of the possibility that ASXL1 truncations might act as gain-of-function mutations of the ASXL1–BAP1 complex, we examined whether hyperactive ASXL–BAP1 complexes could alter the fate of haematopoietic cells *in vivo*. *ASXL1* mutations in myeloid cancers often co-occur with mutations in the *TET2* gene (encoding the 5-methylcyosine oxidase TET2)^24^,^25^. By generating bone marrow chimeras, we demonstrate that the hyperactive ASXL1–BAP1 complex cooperates with loss of TET2 to skew lineage commitment of haematopoietic cells to the myeloid lineage. These results suggest a functional interaction between H2A ubiquitination and the DNA modifications mediated by TET proteins.

## Results

### *ASXL1* truncations enhance activity of the PR-DUB complex

**Figure 1:**
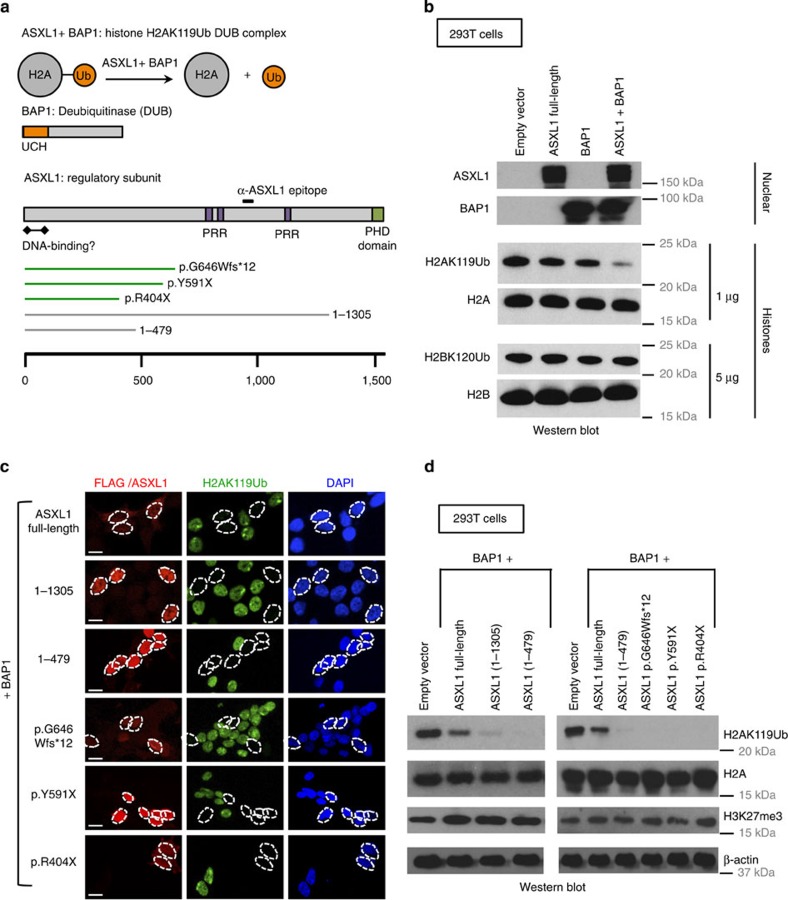
Leukemia-associated ASXL1 truncation mutations cooperate with BAP1 to promote deubiquitination of H2AK119Ub. (**a**) ASXL1 interacts with BAP1 to form a deubiquitinase complex that acts on H2AK119Ub. ASXL1 is the regulatory subunit of this complex and BAP1 is the deubiquitinase. The ubiquitin-carboxyl hydrolase (UCH) domain of BAP1 is at its N terminus. ASXL1 has a C-terminal atypical PHD Zinc-finger, a putative N-terminal DNA-binding domain and three PRRs that are thought to facilitate protein–protein interactions. Shown below are three cancer-associated ASXL1 mutations and two ASXL1 truncations, ASXL1(1–1305), and ASXL1(1–479), which we have employed in our studies. (**b**) 293T cells were mock transfected or transfected with mammalian expression vectors encoding BAP1, full-length ASXL1, or both. Expression of ASXL1 and BAP1 was confirmed by western blotting carried out on nuclear lysates. Acid-extracted histones were probed with antibodies against the indicated proteins and histone modifications. As shown, co-transfection of ASXL1+BAP1 results in marked reduction in levels of H2AK119Ub but not H2BK120Ub. (**c**,**d**) HEK293T cells were co-transfected with mammalian expression vectors encoding BAP1 with full-length ASXL1 or ASXL1 truncations/mutations as indicated. Western blottings to examine expression of ASXL1 and ASXL1 mutations are shown in Supplementary Fig. 1c. (**c**) Cells were fixed 48 h post transfection, permeabilized and stained with anti-ASXL1 (red) or anti-FLAG (red) and anti-H2AK119Ub (green) antibodies. Scale bar, 10 μm. (**d**) Nuclear lysates and acid-extracted histones prepared from transfected cells were subjected to western blotting.

The most prominent variant of *ASXL1* encodes a protein of 1,541 amino acids^26^,^27^, containing an N-terminal region that is a putative DNA-binding domain^28^,^29^, three PRRs that may facilitate interactions with other proteins and an atypical PHD at the C terminus (Fig. 1a).

Mutations of both *ASXL1* and *BAP1* occur frequently in patients with myeloid and other cancers^16^,^30^,^31^,^32^. Of 712 mutations of the *ASXL1* gene classified in the COSMIC (Catalogue of Somatic Mutation in Cancer) database^33^ as associated with haematological cancers, 632 (>88%) give rise to prematurely truncated variants of ASXL1 that retain the N-terminal region of ASXL1 (Supplementary Fig. 1a). *ASXL1* mutations in Bohring–Opitz syndrome are also predicted to give rise to truncated variants^21^,^22^ (Supplementary Fig. 1b). The N-terminal region of ASXL1, specifically amino acids 2–365 of ASXL1, is sufficient to promote the H2AK119Ub DUB activity of BAP1 on nucleosomal templates *in vitro*^15^. To examine whether cancer-associated truncated versions of ASXL1 could promote the catalytic activity of BAP1 in a cell-based assay, we generated mammalian expression vectors for ASXL1(1–1305); C-terminally 3XFLAG-tagged ASXL1(1–479) and three C-terminally 3XFLAG-tagged ASXL1 mutations documented in myelodysplastic syndrome (MDS) patients—p.G646Wfs*12, p.Y591X and p.R404X (Fig. 1a, bottom).

HEK293T cells were transiently transfected with complementary DNAs encoding BAP1 and full-length or truncated ASXL1, and global H2AK119Ub levels were assessed by immunocytochemistry and western blotting (Fig. 1b–d). As attaching an epitope tag to full-length ASXL1, at either the N or C terminus, compromised its ability to support the catalytic activity of BAP1, full-length ASXL1 and ASXL1(1–1305) (PHD-del) were left untagged and were detected with an antibody to an epitope located between the second and third PPRs (see Fig. 1a); all other truncations were tagged with the 3XFLAG tag at the C terminus and detected with an anti-FLAG antibody (Supplementary Fig. 1c). ASXL1(1–1305) was expressed at higher levels and showed increased nuclear localization relative to full-length ASXL1 (Fig. 1c and Supplementary Fig. 1c). Moreover, both ASXL1(1–1305) and ASXL1(1–479) stabilized the expression of BAP1 protein (Supplementary Fig. 1c) and all truncated ASXL1 proteins enhanced the H2AK119Ub-DUB activity of BAP1 in HEK293T cells to a substantially greater extent than observed for full-length ASXL1 (Fig. 1d). Although recent studies suggest that H2AK119Ub has an important role in recruitment of the PRC2 complex^13^, there was no perceptible alteration in global H3K27me3 levels in these transient assays (Fig. 1d), but inactive X chromosomes (Barr bodies) stained strongly for both H2AK119Ub and H3K27me3 as previously observed^34^ (Supplementary Fig. 1d).

### H2AK119Ub depletion skews EML cells to mast cells

We next asked whether we could investigate the biological functions of H2AK119Ub by stably expressing truncated ASXL1–BAP1 complexes in physiologically relevant cell types; based on our data in HEK cells, we expected to see global erasure of the H2AK119Ub modification under these conditions. We chose to use EML cells, a multipotent haematopoietic precursor cell line generated by transducing bone marrow cells isolated from 5-fluorouracil (5-FU)-treated mice with a dominant-negative retinoic acid receptor-α (ref. 35). EML cells are c-Kit^+^ Sca1^+^; they remain multipotent when cultured in media containing stem cell factor (SCF)^35^, but can be differentiated into erythroid, B lymphoid, mast cell or myeloid lineages by addition of erythropoietin, interleukin-7 (IL-7), IL-3 or granulocyte–macrophage colony-stimulating factor (GM-CSF) plus retinoic acid, respectively (Supplementary Fig. 2a).

**Figure 2:**
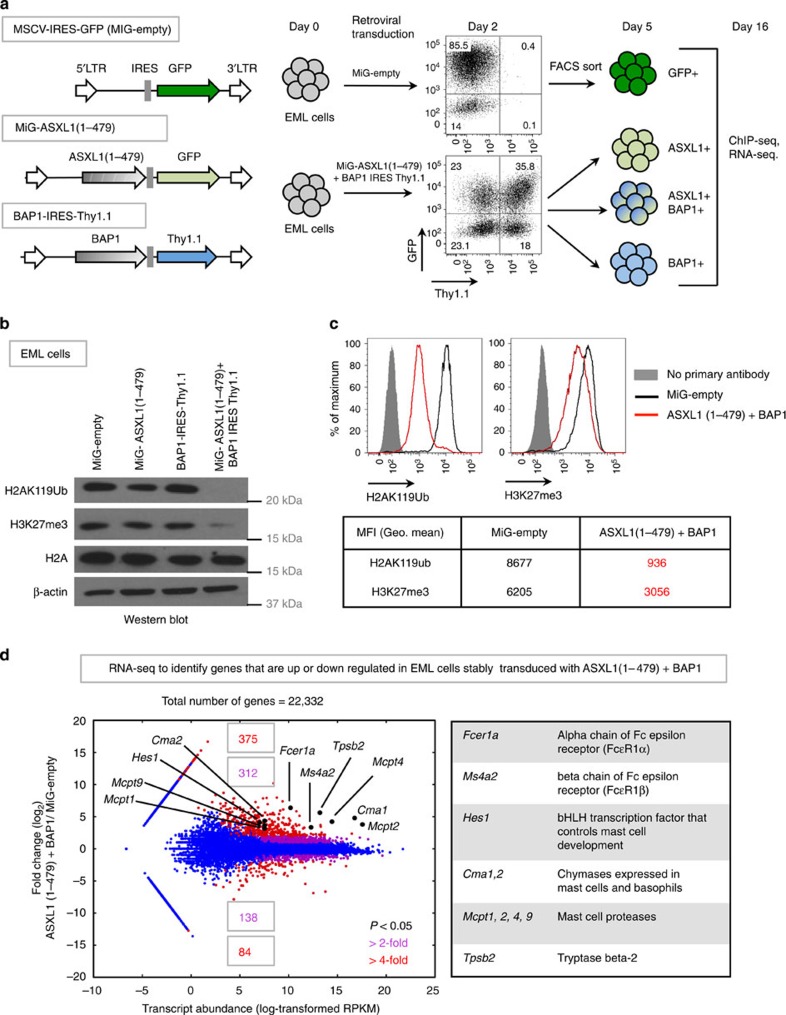
Ectopic expression of ASXL1 truncations+BAP1 results in stable depletion of H2AK119Ub in the EML haematopoietic cell line. (**a**) Using retroviral constructs, we generated EML cell lines that stably express ASXL1(1–479), BAP1, or both. A schematic representation of the retroviral constructs is shown to the left. Based on expression of retroviral reporters, green fluorescent protein (GFP) and Thy1.1, EML cells that express either ASXL1(1–479), BAP1, or both were purified by fluorescence-activated cell sorting (FACS). (**b**,**c**) EML cells were transduced with the indicated viral constructs, FACS sorted and expanded for 16 days in liquid culture in media supplemented with 100 ng ml^−1^ SCF. (**b**) On day 16, nuclear lysates and acid-extracted histones prepared from these cells were subjected to western blotting with indicated antibodies. (**c**) On day 16, cells were subjected to intracellular staining with anti-H2AK119Ub and anti-H3K27me3 antibodies, and levels of H2AK119Ub and H3K27me3 were determined by flow cytometry. Geometric mean fluorescence intensities (gMFI) of H2AK119Ub and H3K27me3 stains were determined by subtracting gMFIs of no primary antibody controls from the absolute gMFIs. (**d**) EML cells transduced with either MiG-empty or ASXL1(1–479)+BAP1 were purified by FACS and expanded for 16 days in liquid culture. On day 16, total RNA was isolated, ribosome-depleted and subjected to RNA-seq. Differential expression and statistical significance were determined using DESeq. Highlighted in the plot and the table are genes that are normally expressed in the mast cell lineage that are upregulated in ASXL1(1–479)+BAP1-transduced EML cells.

EML cells were transduced with empty retrovirus (MiG-empty) or with IRES-GFP and IRES-Thy1.1 retroviruses encoding ASXL1(1–479) (or other ASXL1 mutants) and BAP1, respectively, and the singly and doubly transduced cells were purified by fluorescence activated cell sorting 5 days later (Fig. 2a). Sorted EML cells stably expressing both BAP1 and ASXL1(1–479) showed strong depletion of H2AK119Ub (∼90%), as well as a less dramatic reduction in H3K27me3 (∼50%), as determined by immunoblotting (Fig. 2b and Supplementary Fig. 2b) and flow cytometric analyses (Fig. 2c). Ectopic expression of ASXL1(1–479) alone in EML cells resulted in a minor decrease in H2AK119Ub levels (Fig. 2b and Supplementary Fig. 2c), potentially reflecting cooperation with endogenous BAP1. As transient expression of ASXL1(1–479)+BAP1 in 293T cells did not significantly alter H3K27me3 levels (Fig. 1d), we examined the relative kinetics of loss of H2AK119Ub and H3K27me3 levels in EML cells transduced with ASXL1(1–479)+BAP1 by flow cytometry. Although loss of H2AK119Ub was apparent on day 1 post transduction, loss of H3K27me3 was not apparent till day 2 post transduction (Supplementary Fig. 2d), indicating that sustained expression of hyperactive ASXL1–BAP1 complex is necessary to promote depletion of H3K27me3.

To examine the influence of ASXL1–BAP1 expression on gene transcription, we performed RNA sequencing (RNA-seq) on EML cells transduced with control (MiG-empty) or ASXL1(1–479) and BAP1 encoding retroviruses. Applying criteria of >2-fold change and *P*-value<0.05, we found 687 genes upregulated and 222 genes downregulated in ASXL1(1–479)–BAP1-expressing EML cells relative to control EML cells (Fig. 2d); applying the criterion of >4-fold change, the corresponding numbers were 375 gene upregulated and 84 genes downregulated, respectively. Notably, several of the most highly upregulated genes encoded proteins known to be expressed specifically in mast cells and basophils: *Fcer1a* and *Ms4a2*, encoding the α- and β-chains, respectively, of the Fc epsilon receptor (FcɛR1) that binds IgE; *Cma1* and *Cma2*, encoding mast cell chymases; *Mcpt1*, *2*, *4* and *9*, encoding mast cell proteases; and *Hes1*, a basic helix-loop-helix transcription factor that promotes mast cell differentiation^36^ (Fig. 2d and Supplementary Fig. 2e). The full list of differentially expressed genes can be found in Supplementary Table 1.

**Figure 3:**
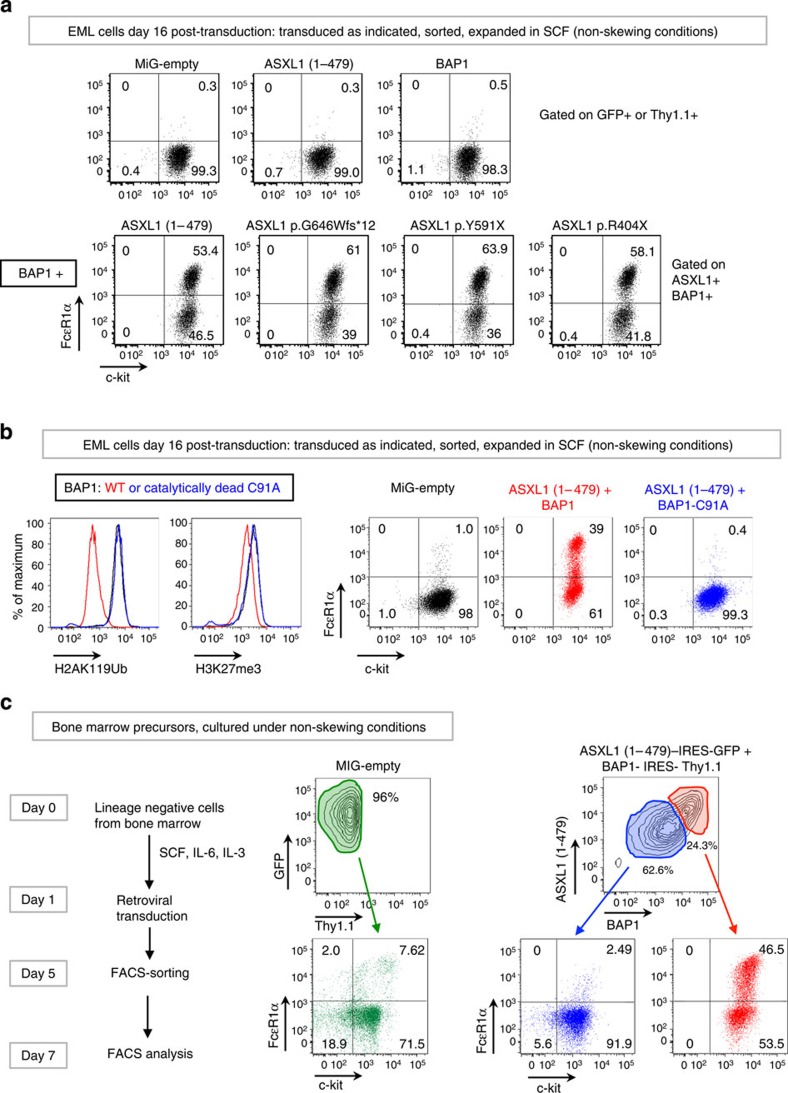
Co-expression of *ASXL1* truncation mutations+BAP1 skews EML and haematopoietic precursor cells to the mast cell lineage. (**a**) EML cells were transduced with retroviral constructs as indicated. Cells were sorted based on expression of green fluorescent protein (GFP) and/or Thy1.1, and expanded in media supplemented with 100 ng ml^−1^ SCF for 16 days post transduction. Commitment to the mast cell lineage was assessed by staining with fluorochrome-conjugated antibodies against c-kit and FcɛR1α. (**b**) EML cells were transduced with retroviral constructs and sorted and expanded as in **a**. In the panels to the left, levels of H2AK119Ub and H3K27me3 were assessed by flow cytometry. Additional controls are shown in Supplementary Fig. 3b. In the panels to the right, commitment to the mast cell lineage was assessed as in **a**. (**c**) Lineage-negative, c-Kit+ (LK) cells isolated from mouse bone marrow (see Supplementary Fig.3c) were transduced and sorted as indicated and expanded in media containing 50 ng ml^−1^ SCF, 10 ng ml^−1^ IL-6 and 10 ng ml^−1^ IL-3 (non-skewing conditions). Commitment to the mast cell lineage was assessed on day 7 by staining with fluorochrome-conjugated antibodies against c-Kit and FcɛR1α.

Consistent with these observations, EML cells transduced with mutant (truncated) ASXL1 proteins and BAP1 showed spontaneous differentiation into c-Kit^+^ FcɛR1α^+^ mast cells by day 16 post transduction (Fig. 3a, bottom panels); individual expression of BAP1 or ASXL1 had no effect (Fig. 3a, top panels, and Supplementary Fig. 3a). BAP1 catalytic activity was essential: co-expression of ASXL1(1–479) with catalytically inactive BAP1-C91A in EML cells did not result in depletion of either H2AK119Ub (as expected) or H3K27me3 (Fig. 3b, left panels) nor did it promote EML cell differentiation to the mast cell lineage (Fig. 3b, right panels). The data indicate that both H3K27me3 depletion and mast cell differentiation are downstream, cell-intrinsic consequences of depletion of H2AK119Ub. The same effect was observed in primary haematopoietic precursor cells: when lineage-negative, c-Kit^+^ (LK) precursor cells isolated from mouse bone marrow (Supplementary Fig. 3b) were transduced with ASXL1(1–479)+BAP1 retroviruses, almost 50% of the resulting ASXL1(1–479)^hi^ BAP1^hi^ cells expressed high levels of c-Kit and FcɛR1α (Fig. 3c).

### H2AK119Ub and H3K27me3 co-occur at repressed genes

**Figure 4:**
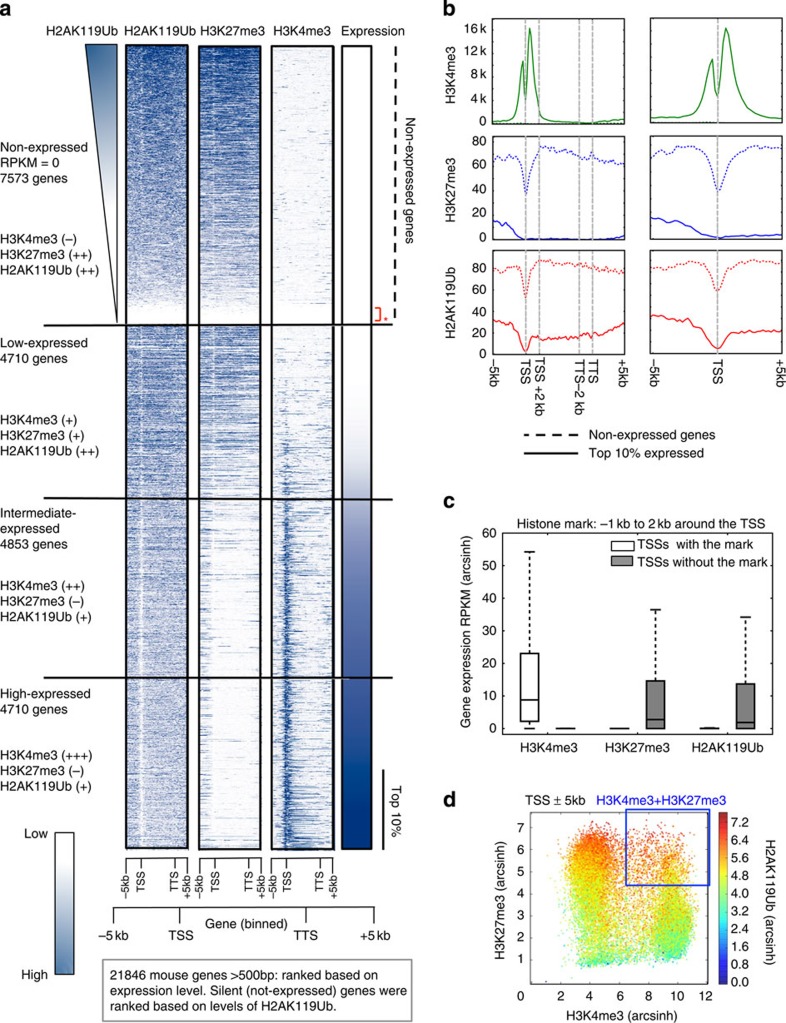
H2AK119Ub marks are present at the expressed and non-expressed genes in EML cells. Mononucleosomes prepared from EML cells transduced with MiG-empty retrovirus were immunoprecipitated with H2AK119Ub, H3K27me3 and H3K4me3 antibodies, and subjected to ChIP-seq. (**a**) Genes were ranked based on levels of expression. Shown alongside is the genome-wide distribution of H2AK119Ub, H3K27me3 and H3K4me3 marks across the gene body and ±5 kb around the gene body of all genes >500 bp in length. Based on expression levels in EML cells, we categorized genes into four categories: non-expressed, low, intermediate and high expressed. Non-expressed genes were ranked based on levels of H2AK119Ub. Areas around TSS (−5 kbp, 0 bp) and around TTS (0 bp, +5k bp) were both divided into twenty-five 200-bp bins. Gene bodies were divided into 50 bins of equal width. Genes <500 bp were excluded due to visualization artefacts caused by gene binning. (**b**) Average input-corrected levels of H2AK119Ub, H3K4me3 and H3K27me3 marks across non-expressed genes and top 10% of genes sorted by expression are represented in two formats, across gene body and ±5 kb around the gene (left) and ±5 kb around TSSs (right). (**c**) Presence of H2AK119Ub, H3K27me3 and H3K4me3 marks was defined based on ≥10% overlap between SICER peak calls and the area −1 kb to +2 kb around the TSSs. RPKM (reads per kilobase per million) values of genes marked by or lacking the said mark are represented as a box showing the 25th, 50th and 75th percentiles, and whiskers extending to 1.5 times interquartile range. (**d**) To determine the overlaps between TSSs marked by the H2AK119Ub, H3K4me3 and H3K27me3, enrichments of H3K4me3, H2AK119Ub and H3K27me3 were calculated with respect to input signal in 200 bp bins ±5 kb around each TSS. Total enrichment for each TSS was determined by summing the enrichment values of the ±5 kb area; H2AK119Ub enrichement was colour coded as indicated on the right.

To examine the relationship between H2AK119Ub, H3K27me3 and transcriptional activity in EML cells, we mapped the genomic distribution of H2AK119Ub, H3K4me3 and H3K27me3 marks by chromatin immunoprecipitation-sequencing (ChIP-seq) on mononucleosomal DNA generated from parental EML cells (MiG-empty-transduced). For H2AK119Ub ChIP analysis, we used a high-affinity antibody, distinct from the EC65 anti-H2AK119Ub antibody used in previous studies^12^,^37^,^38^,^39^; the problems with the EC65 antibody have been discussed elsewhere^15^,^34^,^39^. We ranked protein-coding genes based on expression from lowest to highest and classified them into four categories as follows: not expressed; low expressed (bottom 33% of expressed genes); intermediate expressed (middle 33% of expressed genes); and high expressed (top 33% of expressed genes); Fig. 4a shows results at gene bodies (−5 to+5 kb), Supplementary Fig. 4a shows results centred at the transcription start site (TSS) (−5 to+5 kb) and Fig. 4b shows results averaged and normalized for non-expressed genes and top 10% of expressed genes. As expected^40^,^41^, the H3K4me3 modification showed a bimodal peak centred at the TSS and was effectively absent from non-expressed genes; its levels near the TSS increased with gene expression (Fig. 4a,b and Supplementary Fig. 4a,b). Also as expected^41^,^42^, the H3K27me3 modification showed a generally opposite trend: it was depleted at the TSS, broadly distributed from ±5 kb across the gene body and effectively absent from the TSS and gene bodies of the most highly expressed genes (Fig. 4a,b and Supplementary Fig. 4a,b).

We found that co-occurrence of H2AK119Ub and H3K27me3 correlated strongly with gene repression (Fig. 4a,b and Supplementary Fig. 4a,b), consistent with the notion that H2AK119Ub is a ‘repressive' histone modification associated with poorly expressed genes^12^. The genomic distribution of the H2AK119Ub modification generally resembled that of H3K27me3 (Fig. 4 and Supplementary Fig. 4): almost 74% of genomic regions marked by H2AK119Ub in EML cells also bore H3K27me3 (Supplementary Fig. 4c). In the vicinity of genes, H2AK119Ub was mostly associated with not-expressed and low-expressed genes, but was also present, albeit at considerably lower levels, around the TSS and in the gene body of highly expressed genes (Fig. 4a,b and Supplementary Fig. 4a,b). A small number of silent gene loci were devoid of all three marks—H3K4me3, H3K27me3 and H2AK119Ub (red asterisks in Fig. 4a and Supplementary Fig. 4a). Globally, genes bearing H2AK119Ub at their TSSs showed very low expression compared with genes lacking H2AK119Ub (Fig. 4c), as expected^12^. A plot of H3K4me3 and H3K27me3 levels around the TSS (±5 kb), in which each region is represented by one dot, showed that promoters marked by high H3K27me3 but not H3K4me3 carried the highest absolute levels of H2AK119Ub (red colour), whereas promoters with low H3K27me3 had the lowest levels of H2AK119Ub (blue and green colours; Fig. 4d). Notably, however, H2AK119Ub levels were also high at ‘bivalent' promoters marked by both H3K4me3 and H3K27me3 (ref. 41; Fig. 4d, see demarcated region); this correlation extended beyond promoter/TSS regions, as H2AK119Ub was present at 86% of genomic regions that carried both H3K4me3 and H3K27me3 (Supplementary Fig. 4d).

**Figure 5:**
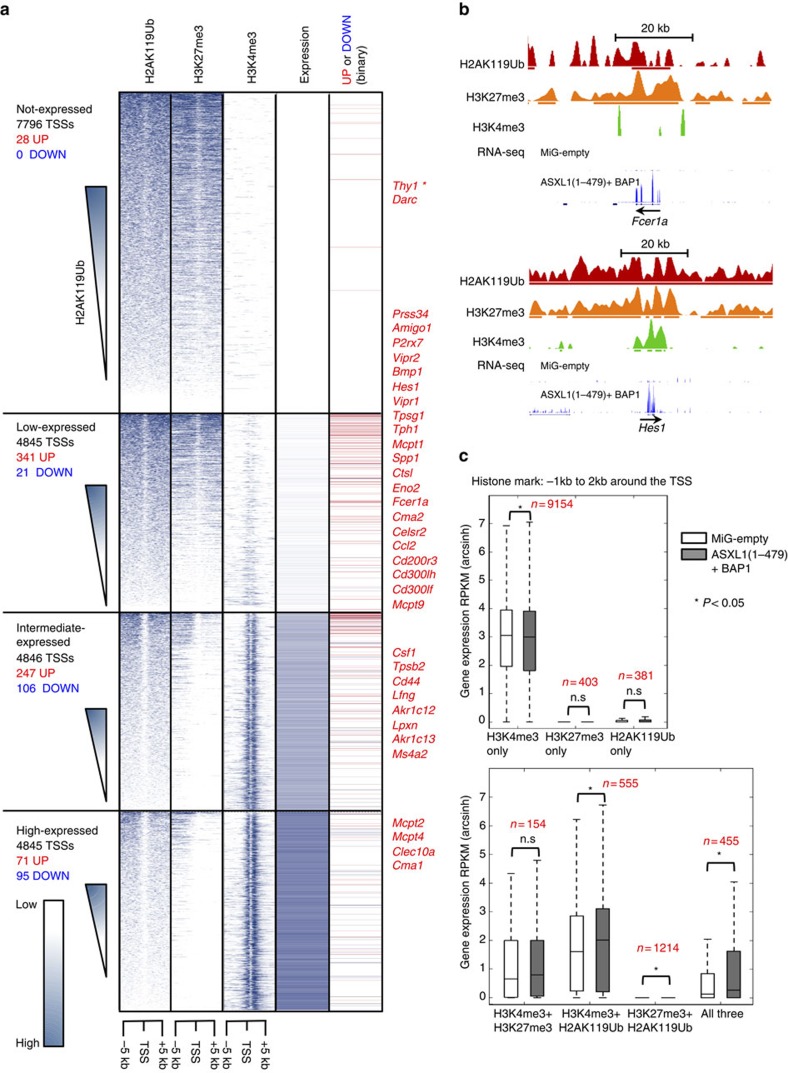
Expression of ASXL1(1–479)+BAP1 leads to upregulation of low-expressed and intermediate-expressed genes. (**a**) Input-corrected distribution of H2AK119Ub, H3K4me3 and H3K27me3 marks ±5 kb around mouse TSSs divided into fifty 200-bp bins is shown alongside gene expression levels in EML cells transduced with MiG-empty. Genes within each sub-class were arranged in descending order of H2AK119Ub levels. In the column to the far right is a binary representation of change in expression at these gene loci on transduction with ASXL1(1–479)+BAP1; genes upregulated or downregulated >2-fold, (*P*<0.05, as determined by DESeq) are represented with red and blue lines, respectively. *Thy1 is marked with an asterisk to highlight the fact that BAP1-expressing retrovirus carried Thy1.1 (a variant of *Thy1*) as the reporter. Names of some upregulated genes (including mast cell-associated genes) are indicated to the right. (**b**) UCSC browser tracks of H2AK119Ub, H3K27me3 and H3K4me3 marks from MiG-empty-transduced EML cells and RNA-seq tracks from both MiG-empty- and ASXL1(1–479)+BAP1-transduced cells of two mast cell genes *Fcer1a* and *Hes1*. The bold lines below the profiles of histone marks represent SICER peak calls. Scale bar, 20 kb. (**c**) The presence of H2AK119Ub, H3K27me3 and H3K4me3 marks in MiG-empty-transduced EML cells −1 to +2 kb around each TSS was determined based on 10% or greater overlap between SICER peak calls. Mean and range of RPKM (arcsinh-transformed) values of genes marked by combinations of histone marks are represented as box and whisker plots as in **c**. Statistical significance was determined by two-tailed Mann–Whitney *U*-test (*P<0.05).

We then examined the changes in gene expression after transduction with ASXL1(1–479) and BAP1 (Fig. 5a,b and Supplementary Fig. 5a,b). Overall, the genes that were upregulated in H2AK119Ub-depleted cells (including mast cell genes) were already expressed at low or intermediate levels in the parental (MiG-empty-transduced) EML cells; repressed genes, which lacked H3K4me3 and were marked only by H2AK119Ub and H3K27me3 at their promoters in parental MiG-empty-transduced EML cells, remained repressed after H2AK119Ub depletion (Fig. 5a and Supplementary Fig. 5a). In fact, only 28 out of 7,796 (fewer than 0.4%) of all repressed genes were upregulated in H2AK119Ub-depleted EML cells (Fig. 5a). In general, genes significantly upregulated in ASXL1(1–479)+BAP1-transduced cells relative to MiG-empty-transduced EML cells were genes whose promoters were marked with (i) both H2AK119Ub and H3K4me3; (ii) both H2AK119Ub and H3K27me3; or (iii) all three modifications, H3K4me3, H2AK119Ub and H3K27me3 (Fig. 5c). Genes whose promoters bore H2AK119Ub alone or both H3K4me3 and H3K27me3 also showed an increase, which however did not reach statistical significance (*P*-values=0.074 and 0.41, respectively, determined using the two-tailed Mann–Whitney *U*-test). Moreover, similar to genes whose promoters bear the dual H3K4me3/H3K27me3 modification^43^,^44^, genes whose promoters bear H2AK119Ub and at least one of the other two modifications are more likely to be upregulated on ASXL1–BAP1 expression and consequent H2AK119Ub depletion (Fig. 5c). These data show clearly that the presence of H2AK119Ub at promoter/TSS regions exerts a repressive influence on gene expression, but that loss of H2AK119Ub through co-expression of ASXL1–BAP1 does not by itself impair repression of silenced gene loci.

### ASXL1–BAP1 expression promotes expansion of myeloid cells

**Figure 6:**
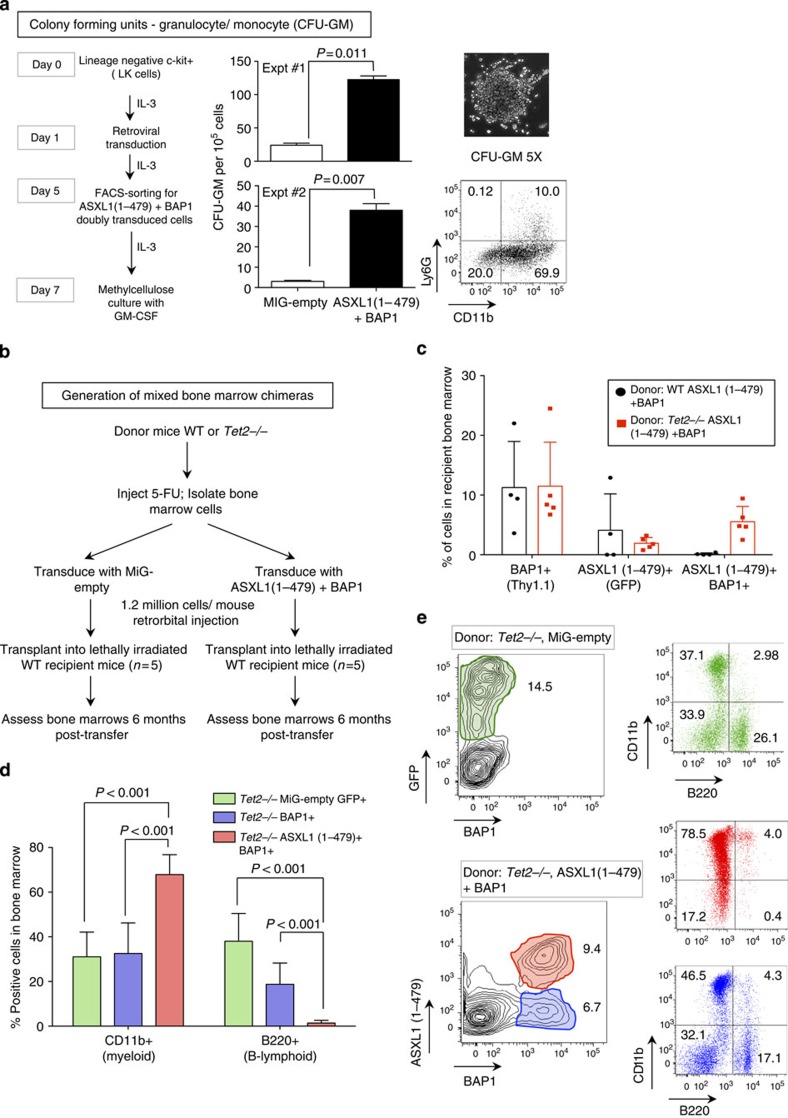
ASXL1 truncations synergize with BAP1 and TET2 loss-of-function to skew commitment to the myeloid lineage *in vivo*. (**a**) Lineage-negative, c-Kit+ (LK) cells isolated from mouse bone marrow and transduced with either MiG-empty or ASXL1(1–479)+BAP1. Transduced cells were expanded in media supplemented with 10 ng ml^−1^ IL-3, fluorescence-activated cell sorted (FACS) for Thy1.1 and green fluorescent protein (GFP) expression on day 5, and expanded in liquid medium supplemented with 10 ng ml^−1^ IL-3 till day 7. On day 7, the cells were washed extensively to remove traces of IL-3. Cells (10^5^) were plated in triplicate in 35-mm dishes in methylcellulose medium supplemented with 10 ng ml^−1^ GM-CSF. Numbers of CFU-GM were counted on day 14. Statistical significance was determined by two-tailed *t*-test. Representative image and FACS profile of a CFU-GM are shown to the right. (**b**) 5-FU-treated bone marrow cells were harvested from WT and TET2-deficient donor mice as described in Methods. The donor cells were transduced as indicated 24 h post transduction; 1.2 × 10^6^ cells were transplanted into irradiated recipient mice by intravenous injection. (**c**) The bone marrow was harvested from recipient mice 6 months post transfer. Transduced cells were identified based on expression of GFP and Thy1.1 reporters. Shown are percentages of BAP1, ASXL1(1–479) and doubly transduced cells in the bone marrows of recipient mice 6 months post transfer. Each dot represents one mouse. (**d**) Percentages of CD11b (myeloid)- and B220 (B-lymphoid)-positive cells that were transduced with MiG-empty, BAP1 alone or ASXL1(1–479)+BAP1 retroviruses were determined by flow cytometry. Data shown are from recipients that received cells from TET2-deficient donors; percentages represent mean±s.d. from five recipients. Statistical significance was determined by two-tailed *t*-test; *n*=5. (**e**) Representative FACS plots of CD11b- and B220-positive cells in the bone marrow of recipient mice that were transplanted with *Tet2*^*−/−*^ bone marrow cells 6 months post transfer.

Although *ASXL1* mutations have been documented in mastocytosis patients^45^, the majority of *ASXL1* mutations are observed in myeloid cancers^16^. Our studies here raise the possibility that *ASXL1* mutations may act as gain-of-function mutations of the ASXL1–BAP1 complex. To examine whether hyperactive ASXL1–BAP1 complexes could skew the differentiation of bone marrow precursor cells to granulocyte–macrophage (GM) lineages, we plated control MiG-empty-transduced and ASXL1(1–479)+BAP1-transduced LK cells in methylcellulose media supplemented with GM-CSF (granulocytic macrophage colony-stimulating factor) (Fig. 6a). In two independent experiments, ASXL1 (1–479)+BAP1 markedly enhanced the expansion of precursor cells (CFU-GM, colony-forming units that give rise to cells of the GM lineage) by >5-fold (Fig. 6a).

To examine whether ectopic expression of ASXL1(1–479)+BAP1 could skew differentiation to the myeloid lineage *in vivo*, we generated mixed bone marrow chimaeras. Lethally irradiated C57BL/6 recipient mice were transplanted with wild-type (WT) bone marrow, either transduced with MiG-empty retrovirus (GFP-marked) or subjected to combined infection with ASXL1(1–479)-IRES-GFP+BAP1-IRES-Thy1.1 retroviruses (Fig. 6b and Supplementary Fig. 6a). Six months post transfer, we did not detect any doubly transduced (GFP^+^ Thy1.1^+^) cells in the bone marrow of recipient mice that received WT donor cells transduced with ASXL1(1–479) and BAP1 retroviruses (Fig. 6c and Supplementary Fig. 6a, top panels).

Given that *TET2* mutations frequently co-occur with *ASXL1* mutations in patients with myeloid malignancies^46^,^47^, we hypothesized that a second mutation (for example, in *Tet2*) might be necessary for optimal survival, proliferation and/or expansion of the doubly transduced bone marrow precursor cells. We therefore repeated the ASXL1(1–479)+BAP1 transduction experiment using donor cells derived from *Tet2*-deficient mice^48^, which show increased expansion of haematopoietic stem/precursor cells^48^,^49^,^50^. *Tet2*^*−/−*^ donor cells were transduced with either MiG-empty retrovirus or retroviruses encoding ASXL1 (1–479) and BAP1, and transplanted into lethally irradiated recipients (Supplementary Fig. 6a, bottom panels). Six months post transfer, we detected ASXL1(1–479)+BAP1 doubly transduced *Tet2*^*−/−*^ donor cells, but not doubly transduced WT cells, in the bone marrow of recipient mice (Fig. 6c and Supplementary Fig. 6a, right panel). WT and *Tet2*^*−/−*^ donor cells expressing BAP1 alone were detected in all recipients, emphasizing the specificity of the effect; WT and *Tet2*^*−/−*^ cells expressing ASXL1(1–479) alone were detected in some but not all recipient mice (Fig. 6c). Therefore, MiG-empty-transduced *Tet2*^*−/−*^ cells and BAP1-only-transduced *Tet2*^*−/−*^ deficient cells served as external and internal controls to study the fates of doubly transduced cells.

Although BAP1-only-transduced and MiG-empty-transduced *Tet2*^*−/−*^ donor cells gave rise to both Cd11b^+^ (myeloid) and B220^+^ (B cell, lymphoid) cells in the bone marrow of recipient mice (Fig. 6d,e, blue and green bars and plots, respectively), doubly transduced cells were significantly skewed towards the myeloid lineage (Fig. 6d,e, red bars and plots). Doubly transduced *Tet2*^*−/−*^ donor cells also showed increased expression of c-Kit (Supplementary Fig. 6b), indicating that ASXL1(1–479) and BAP1 synergized with TET2 loss-of-function to enhance expansion of TET2-deficient c-Kit^+^ precursor cells in the bone marrow. We consistently documented the presence of doubly transduced cells in the bone marrow of recipients that received ASXL1(1–479)+BAP1-transduced *Tet2*^*−/−*^ donor cells (Fig. 6c–e and Supplementary Fig. 6b). Taken together, our data indicate that the ASXL1 truncations documented in myeloid cancers may cooperate with BAP1 and TET2 loss-of-function, to alter lineage commitment and expansion of haematopoietic precursor cells in the bone marrow.

## Discussion

We have examined the role of H2AK119Ub in regulating gene expression by using hyperactive mutations of the ASXL1–BAP1 complex that allowed us to deplete ∼90% of total H2AK119Ub. Three major points emerge. First, ASXL1–BAP1-dependent depletion of H2AK119Ub was accompanied in EML cells by ∼50% reduction in bulk levels of H3K27me3 and by enhanced differentiation to the mast cell lineage; both effects were entirely dependent on the catalytic activity of BAP1, indicating that global depletion of H2AK119Ub is the primary cause of both the skewed differentiation and the loss of H3K27me3. As in EML cells, ASXL1–BAP1 co-expression promoted the differentiation of primary bone marrow precursor cells to the mast cell lineage *in vitro*. Second, depletion of H2AK119Ub in EML cells did not result in global activation of repressed gene loci—including several *Hox* genes—whose promoters lacked H3K4me3 but were marked by H3K27me3 and H2AK119Ub; instead, we noted a more limited consequence of increased expression of certain genes (including mast cell-specific genes) whose promoters were marked by H2AK119Ub and H3K4me3 or all three modifications H2AK119Ub, H3K4me3 and H3K27me3. Third, in bone marrow chimeras *in vivo*, the hyperactive ASXL1–BAP1 complex cooperated with loss of TET2 to promote commitment of haematopoietic cells to the myeloid lineage, consistent with the fact that combined mutations of *ASXL1* (encoding truncated versions of ASXL1 protein) and *TET2* (encoding loss-of-function mutants) are frequently observed in myeloid disorders^46^,^47^ including MDS, myeloproliferative neoplasms, systemic mastocytosis, chronic myelomonocytic leukemia and acute myeloid leukemia.

At least three previous studies used the problematic EC65 anti-H2AK119Ub antibody^15^,^34^,^39^ to map the distribution of H2AK119Ub in mammalian cells: genome-wide ChIP-seq in mouse embryonic fibroblasts (MEF) 37 and HEK293 T cells^12^, respectively, and ChIP-chip for H2AK119Ub-marked promoters in mouse embryonic stem cells (mESCs)^38^. Kallin *et al.*^37^ mapped H2AK119Ub distribution in control MEFs and MEFs that lacked the PRC1 subunit PCGF4 and restricted their analysis to regions where the presence of H2AK119Ub was dependent on PCGF4 (that is, genomic regions with equivalent levels of H2AK119Ub in control MEFs and PCGF4-deficient MEFs were excluded from the analysis). They found that PCGF4-dependent H2AK119Ub-marked regions overlapped poorly with regions marked by H3K27me3 at both promoter and non-promoter regions^37^. Gao *et al.*^12^ showed that genes marked by H2AK119Ub were also bound by components of the PRC1 complex, and that some of these gene loci also carried H3K27me3 marks. Here we have used a rigorously tested high-affinity, high-specificity anti-H2AK119Ub antibody in a physiological cell type, EML haematopoietic precursor cells, to examine the relation between H2AK119Ub depletion and changes in gene expression and cell fate. Among other findings, we document a considerable overlap (∼74%) between genomic regions marked by H3K27me3 and H2AK119Ub, consistent with our current understanding of PRC1 and PRC2 recruitment and functions^8^.

We found that depletion of H2AK119Ub in EML cells expressing ASXL1 and BAP1 results in significant upregulation of low-expressed and intermediate-expressed genes originally marked by H2AK119Ub. More precisely, depletion of H2AK119Ub led to targeted upregulation of genes whose TSSs were marked in parental EML cells by H3K4me3 and H2AK119Ub, or with all three modifications H3K4me3, H3K27me3 and H2AK119Ub. Our data are in agreement with a previous study showing that depletion of H2AK119Ub in mESC resulted in de-repression of ‘bivalent' gene loci marked by both H3K4me3 and H3K27me3 marks^43^. This study used tamoxifen-inducible deletion of RING1B, the E3 ligase that deposits H2AK119Ub in mESC, to demonstrate that loss of RING1B led to depletion of H2AK119Ub, increased recruitment of RNA Pol II and higher rates of transcription at ‘bivalent' TSSs that carry both H3K27me3 and H3K4me3 modifications in mESC, including *Cdx2*, *Nkx2.9* and *Gata4*. Together, their data and ours support the notion that H2AK119Ub plays an essential role in limiting the recruitment of RNA Pol II to TSSs marked by H3K4me3 and H3K27me3—we document here that these TSSs are also marked by H2AK119Ub.

Mutations or deletions in genes encoding Polycomb group proteins result in mis-expression of *hox* genes in *Drosophila* larvae^51^. Studies of Polycomb proteins in mice have confirmed that both PRC1 and PRC2 proteins play essential roles in repression of *Hox* gene loci in mESC^51^. It is therefore intriguing that *Hox* gene repression remained unchanged in H2AK119Ub-depleted EML cells relative to controls. Nonetheless, our findings are in line with previous studies by Bickmore and colleagues^52^, who demonstrated that the ability of the PRC1 complex to repress expression of *Hox* genes in mESC was independent of the E3 ligase activity of RING1B.

ASXL1 has been reported to interact directly with EZH2 and EED, catalytic and scaffold subunits, respectively, of the PRC2 methyltransferase complex that deposits H3K27me3; moreover, short hairpin RNA-mediated depletion of ASXL1 in a human leukemia cell line resulted in a decrease in bulk H3K27me3 levels and loss of the H3K27me3 mark at multiple genomic loci^26^. The authors suggested that in addition to functioning as the obligate regulatory subunit of the ASXL1–BAP1 complex, ASXL1 might also directly regulate the deposition and/or removal of H3K27me3. We note that unbiased co-immunoprecipitation experiments to identify BAP1- and Calypso-interacting proteins^15^,^19^,^53^ did not identify EZH2 or its *Drosophila* homologue as partners. It is possible that in mammalian cells ASXL1 is a component of two distinct complexes, one that comprises ASXL1 and BAP1, and a second complex that comprises ASXL1 and components of the PRC2 complex. Notably, deletion of ASXL1 results in reduced bulk levels of H3K4me3 (ref. 54), although how ASXL1 modulates levels of H3K4me3 is unclear.

Here we document that the ability of the hyperactive ASXL1–BAP1 complex to deplete H3K27me3 was contingent on the catalytic activity of BAP1. Therefore, we propose that loss of H3K27me3 in EML cells expressing hyperactive ASXL1–BAP1 complexes is a direct consequence of loss of H2AK119Ub. This is supported by two recent studies demonstrating that H2AK119Ub deposited by variant PRC1 complexes plays an essential role in recruitment of H3K27me3-depositing PRC2 complexes^13^,^55^. Although the hyperactive ASXL1–BAP1 complex clearly targets H2AK119Ub, our studies do not rule out the possibility that it also removes ubiquitin from some other substrate(s) to promote loss of H3K27me3, diminishes the multipotency of EML cells and/or alters their propensity to differentiate.

Whether the truncated ASXL1 proteins expressed in cancers and developmental disorders are functional or not remains a contentious question. Although some studies have proposed that ASXL1 truncations represent loss-of-function mutations^26^,^54^,^56^, expression of the ASXL1 frameshift mutation p.E635Rfs*15 in otherwise WT mouse bone marrow cells promoted the development of MDS-like symptoms, albeit with prolonged latency, supporting the idea that these truncations may in fact be functional^27^. Studies on *Drosophila* Asx have also not provided a clear answer. Müller and colleagues^15^,^57^ used two Asx-mutant *Drosophila* lines in their studies of the ASX-Calypso complex, *Asx*^*22P4*^ and *Asx*^*27J6*^. (i) *Asx*^*22P4*^ is an Asx-null allele^15^ and *Asx*^*22P4*^ embryos fail to express detectable amounts of Asx, although there is no mutation within the *Asx* open reading frame (ORF). Embryos homozygous for this *Asx*^*22P4*^-null mutation do not express detectable levels of Asx and protein levels of Calypso are also significantly diminished, indicating that these embryos lack not only Asx but also a functional Asx-Calypso complex. Consistent with this finding, *Asx*^*22P4*^ mutant embryos show clear loss-of-function of Asx-Calypso DUB activity as judged by an increase in bulk levels of H2AK118Ub (corresponding to mammalian H2AK119Ub). (ii) *Asx*^*27J6*^ carries a frameshift mutation that results in expression of a truncated protein, Asx(1–432), followed by 15 additional amino acids resulting from the frameshift^15^. In embryos homozygous for the *Asx*^*27J6*^ mutation, both the truncated Asx protein and Calypso are expressed at levels comparable to WT embryos^15^. However, Müller and colleagues^15^ did not evaluate the levels of H2AK118Ub in *Asx*^*27J6*^ mutant embryos where the truncated Asx protein and Calypso are both detectably expressed. Based on our results, we postulate that embryos homozygous for *Asx*^*27J6*^ mutation express a hyperactive Asx-Calypso complex, and that levels of H2AK118Ub in these embryos are significantly lower than in WT embryos—this awaits verification. In fact, *Drosophila* embryos that lack Sce^58^, the *Drosophila* RING homologue that deposits H2AK118Ub, also show a similar loss of Polycomb repression and posterior transformation including mis-expression of Ubx in the wing disc. Therefore, we propose that *Asx*^*27J6*^ mutations and loss of Sce lead to posterior transformation by the same mechanism—by promoting loss of H2AK118Ub. Further studies on *Drosophila* embryos that are homozygous for *Asx*^*27J6*^ mutation and express a truncated Asx(1–432) would be vital in clarifying whether N-terminal truncations of Asx and mammalian ASXL family proteins act as gain-of-function mutations of the PR–DUB complex or not.

The *ASXL1* mutations associated with myeloid malignancies^16^ and in Bohring–Opitz syndrome^21^,^22^ are heterozygous mutations that result in deletion of either the PHD domain at the very C terminus, or more frequently, deletion of both the PHD domain and all three PRR domains. Heterozygous *de novo* germline mutations that result in truncations that lead to loss of the C-terminal region of ASXL3 (ref. 23) have also been documented in the Bohring–Opitz syndrome; similarly, heterozygous *ASXL2* mutations that would result in premature truncations have been reported in pediatric acute myeloid leukemia cases^20^. We have shown that in HEK293T cells full-length human ASXL1(1–1541) is present in both the nucleus and the cytoplasm, whereas ASXL1(1–1305), which lacks the PHD domain, and other ASXL1 truncations that lack both PHD and PRR domains, are exclusively nuclear and expressed at higher levels. These data suggest that the C-terminal regions of ASXL family proteins may have an autoinhibitory function. Given that *ASXL1*, *ASXL2* and *ASXL3* mutations are invariably heterozygous^16^, our studies raise the possibility that the truncated versions of ASXL1 mutations in cancers and in Bohring–Opitz syndrome could act as gain-of-function mutations. Alternatively, truncated ASXL1 proteins might function in a dominant-negative manner, by interfering with the expression, activity or other properties of WT ASXL1. Future studies using an *Asxl1* ‘knock-in' mouse model that would allow conditional heterozygous deletion of the C terminus of ASXL1 will be needed to carefully address this question.

## Methods

### Plasmids

Mammalian cytomegalovirus (CMV) promoter (pCMV)-driven plasmids that encode full-length human BAP1 and ASXL1 were purchased from Origene (Rockville, MD). pCMV and MSCV-IRES-GFP (MiG) vectors encoding ASXL1 truncations 1–1,305, 1–479 and p.R404X were generated by PCR amplification/restriction digestion and sub-cloning. We purchased a custom DNA template encoding *ASXL1* p.G646Wfs*12 from Genescript (Piscataway, NJ), which harboured two silent mutations that eliminated two internal EcoRI sites in the DNA sequence. This DNA sequence was used as template to generate vectors encoding C-terminally FLAG-tagged *ASXL1* p.G646Wfs*12 and p.Y591X. BAP1 was PCR-amplified and cloned into pDEST-IRES-Thy1.1 using the Gateway system, with reagents purchased from Life Technologies (Grand Island, NY). All primers used in this study were obtained from Integrated DNA Technologies (Coralville, IA).

### Reagents and mice

H3K4me3 (9,727), H3K27me3 (9,733) and H2AK119Ub (8,240) antibodies were purchased from Cell Signaling Technologies (Danvers, MA). Antibodies against H3K27me3 (ab6002), ASXL1 (ab50817) and BAP1 (05–671) were purchased from Abcam (Cambridge, UK) and EMD Millipore (Billerica, MA), respectively. Anti-FLAG M2 (F3165) antibody was obtained from Sigma-Aldrich (St Louis, MO). Horseradish peroxidase-conjugated anti-mouse (7,076, 1:20,000) and anti-rabbit (A545, 1:20,000) secondary antibodies for western blotting were obtained from Cell Signaling Technology and Sigma-Aldrich, respectively. All primary antibodies were diluted to 1 in 1,000 in 10% BSA prepared in PBS (1 × PBS) for Western Blotting. Anti-H3K27me3 antibody (ab6002, Abcam) was only used for immunocytochemistry shown in Supplementary Fig. 1C; anti-H3K27me3 (9,733, Cell Signaling Technologies) antibody was used for all other experiments. For flow cytometric analyses, fluorochrome-conjugated antibodies against c-Kit (eBioscience (San Diego, CA), 25–1171, 1:200), CD11b (clone M1/10, eBioscience, 14–0112, 1:200), Ly6G (clone 1A8, BioLegend (San Diego, CA), 1,27,607, 1:200), FcɛR1α (clone MAR-1, eBioscience, 17-5,898, 1:200) and Thy1.1 (clone OX-7, BioLegend, 2,02,523, 1:500) were used. Murine SCF (250-03), IL-3 (213-13) and IL-6 (216-16) were purchased from Peprotech (Rocky Hill, NJ). Anti-rabbit Alexa Fluor-647 (A-21244), anti-rabbit Alexa Fluor 488 (A-11,008) and mouse anti-mouse Alexa Fluor 594 (A-11,032) secondary antibodies for immunostaining and intracellular staining for flow cytometry were purchased from Life Technologies. Methylcellulose medium (HSC001) was purchased from R&D Systems (Minneapolis, MN). C57BL/6 mice were purchased from Jackson Labs (Bar Harbor, ME). The *Tet2*^*−/−*^ strain of mice used in this study has been previously described^48^. The mice were housed in pathogen-free animal facility in the La Jolla Institute for Allergy and Immunology, and were used in accordance with protocols approved by the Institutional Animal Care and use Committee.

### Transfection and cell culture

293T and Plat-E cells were purchased from American Type Culture Collection (ATCC, Manassas, VA) and Cell Biolabs Inc. (San Diego, CA), respectively. EML cells were a generous gift from Dr Schickwann Tsai (University of Utah). 293T, Plat-E and EML cells were maintained in culture conditions recommended by the ATCC. 293T cells and Plat-E cells were transfected using TransIT-LT1 (MIR 2304) purchased from Mirus Bio LLC (Madison, WI). Ecotropic viruses encoding ASXL1 truncations and BAP1 were generated by transfecting Plat-E cells^59^ with the corresponding constructs. Viral supernatants were collected at 24 and 48 h post transfection and supplemented with Polybrene (AL-118, Sigma-Aldrich) to a final concentration of 4 μg ml^−1^. Both EML cells and primary LK cells were transduced by centrifugation with the viral supernatants for 90 min at 2,000*g* in a centrifuge pre-warmed to 32 °C.

### Bone marrow cell cultures

Bone marrow cells were flushed out of the femurs and tibias isolated from humanely euthanized C57BL/6 mice. LK cells were isolated using mouse lineage-cell depletion kit (Miltenyi Biotec, Germany) followed by fluorescence-activated cell sorting to positively select c-Kit+ cells and to ensure depletion of CD11b^+^ and FcɛR1α^+^ cells (Supplementary Fig. 3B), and cultured overnight in 50 ng ml^−1^ SCF, 10 ng ml^−1^ IL-3 and 10 ng ml^−1^ IL-6, and transduced the following day (day 1). The cells were sorted on day 5 based on expression ofgreen fluorescent protein and Thy1.1. Commitment to the mast cell lineage was assessed based on expression of c-Kit and FcɛR1α on day 7.

### Methylcellulose cultures

For promoting expansion of transduced GM precursors, LK cells were isolated as indicated above, but were cultured overnight in media supplemented with only 10 ng ml^−1^ IL-3. Cells were transduced and sorted as indicated above and maintained in 10 ng ml^−1^ IL-3 for 2 more days. On day 7, the cells were washed extensively to remove residual IL-3. Cells (10^5^) were plated in triplicate in 35-mm dishes in 1.2% methylcellulose media supplemented with 10 ng ml^−1^ GM-CSF. Numbers of CFU-GM were counted 1 week after plating. After counting, the cells were re-suspended in 1 × PBS and stained with antibodies against CD11b and Ly6G, to verify commitment to the GM lineage.

### Immunostaining

For immunostaining, HEK293T cells were cultured and transfected in eight-chamber slides purchased from BD Bioscience (San Jose, CA). Cells were fixed with 4% formaldehyde for 15 min at room temperature and blocked with 5% fetal bovine serum and 0.2% Triton-X 100 in 1X PBS for 1 h at room temperature. Staining was carried out by incubating overnight with primary antibodies (1: 500) in 1% BSA and 0.2% Triton-X 100 in 1X PBS. The slides were rinsed three times with 1X PBS and incubated with fluorochrome-conjugated secondary antibodies (1:1,000) in 1% BSA and 0.2% Triton-X 100 for 1 h at room temperature. The slides were rinsed three times with 1X PBS, air-dried. The cells were coated with ProLong Gold antifade mounting medium with DAPI (4',6-diamidino-2-phenylindole, 100 ng/ml) (Life Technologies, P-36931), overlaid with coverslips and the mounting medium was allowed to cure at least overnight before imaging. Images were acquired on an LSM 710 laser-scanning confocal microscope.

### Intracellular staining for H2AK119Ub and H3K27me3

Staining was carried out in 96-well U-bottom plates and all washing steps were carried out by centrifugation. Cells were fixed in 2% formaldehyde for 15 min at room temperature, washed with 1X PBS and then blocked with 5% fetal bovine serum, 0.2% Triton X-100 supplemented with 20% Fc Block (generated in-house by collecting supernatants from 2.4G2 hybridoma cells) for 1 h at room temperature. Staining was carried out by incubating cells with primary antibody (1: 200) for 1 h in antibody incubation buffer (1% BSA and 0.2% Triton X-100, 20% Fc Block) at room temperature. Cells were washed and then incubated with fluorochrome-conjugated secondary antibodies (1:500) and incubated for 1 h in antibody incubation buffer at room temperature. Cells were washed and the samples were run on the flow cytometer the same day.

### RNA-seq and validation

Total cellular RNA was isolated using Qiaquick RNA isolation kit purchased from Qiagen (The Netherlands). Ribosomal depletion was carried out using the Ribo-zero gold magnetic kit purchased from Epicentre Biotechnologies (Madison, WI). RNA-seq libraries were prepared using SOLiD Total RNA-seq kit purchased from Life Technologies. The quality of the RNA-seq libraries was assessed using the RNA 6,000 Pico kit purchased from Agilent Technologies (San Diego, CA). Sequencing runs were carried out at the LIAI Sequencing Center on a 5,500 SOLiD Sequencer instrument. RNA-seq data represent pooled results from two technical replicates. Real-time reactions were run using SYBR-green mastermix purchased from Roche (Switzerland).

### Preparation of mononucleosomes

Mononucleosomes were prepared using EZ-Zyme chromatin prep kit purchased from EMD Millipore, as per the manufacturer's directions. Briefly, the cells were washed with 1 × PBS to remove traces of media, dounced on ice by resuspending them for 5 min in 10 mM Tris-Cl (pH 8.0), 10 mM NaCl, 3 mM MgCl_2_ and 0.25% Nonidet P-40 supplemented with Complete Protease Inhibitor (Roche). The nuclei were precipitated by centrifugation at 2,000*g* for 10 min and extensively washed in 10 mM Tris-Cl (pH 8.0), 10 mM NaCl and 3 mM MgCl2 to remove traces of detergent and resuspended in EZ-Zyme digestion buffer, and incubated with 1 Unit of EZ-Zyme for 12–15 min (both were determined by careful titration). Digestion was stopped by addition of EZ-Zyme STOP buffer. The samples were centrifuged at 13,000*g*, to remove insoluble material and the nucleosomes in the soluble fraction were used for ChIP-seq experiments. MNase digestion for each sample used for ChIP-seq was verified by running a small aliquot of the nucleosome prep on a 1.5% agarose gel.

### Chromatin immunoprecipitation-sequencing

For anti-H2AK119Ub, anti-H3K4me3 and H3K27me3 ChIP-seqs, nucleosomes were generated from six different EML frozen cell pellets, processed for ChIP as independent samples in duplicate and barcoded with six distinct barcodes for sequencing. Briefly, the nucleosomes were incubated overnight with corresponding antibodies at 4 °C in 10 mM Tris-Cl (pH 8.0), 100 mM NaCl, 1 mM EDTA and 0.1% Triton X-100 (IP buffer). Antibody-bound mononucleosomes were enriched by addition of protein-A DYNAL magnetic beads (Life Technologies) and incubating the mixture for 1 h at 4 °C. The beads were washed 3 × with IP buffer and the antibody–mononucleosome complex was eluted in 1% SDS and 100 mM NaHCO_3_. The eluted nucleosomes were digested with Proteinase K and the DNA was purified using Qiagen min-elute columns and eluted in 12 μl of nuclease-free water. The DNA was quantified using Qubit high-sensitivity reagents (Invitrogen) and 10–30 ng of DNA was used to construct sequencing libraries using ChIP-seq library preparation kits for SOLiD sequencing purchased from New England Biolabs (Ipswich, MA). The quality of the libraries were assessed on Agilent 2100 Bioanalyzer using the Agilent high-sensitivity DNA kit purchased from Agilent Technologies. Anti-H2AK119Ub, anti-H3K4me3 and H3K27me3 ChIP-seq runs were carried out at the LIAI Sequencing Center on a 5500 Solid Sequencer instrument.

### ChIP-seq data analysis

Sequenced reads were aligned using Bowtie (version 0.12.8)^60^, allowing only uniquely mapping reads, fragment sizes of 90–250 bp, and removing duplicates. Read density profiles were generated with WIGGLER ( https://code.google.com/p/align2rawsignal/). When calculating enrichments of areas around TSS, pseudo-count of 1 was added to both signal and input values. We employed a spatial clustering approach for identification of ChIP-enriched regions (SICER)^61^ to identify regions of the genome enriched for H2AK119Ub, H3K4me3 and H3K27me3 marks in EML cells (FDR=0.01), and used the SICER peak-calls to identify the overlap between regions of the genome that carried H3K4me3, H3K27me3 and H2AK119Ub marks. For H3K4me3 and H3K27me3 the parameters were as suggested in the SICER documentation and for H2AK119Ub window size 800 bp and gap size 2400, bp were used.

### RNA-seq analysis

Differential expression cells were obtained by aligning the data using TopHat (version 2.0.9)^62^. Quantification of gene-wise read counts were obtained using HTSeq ( http://www-huber.embl.de/users/anders/HTSeq) and then using DESeq^63^. eXpress^64^ was used to obtain transcript level expression estimates. Among possible several transcripts per gene, only one was selected by taking the one with the highest expression estimate and this transcript was also used when analysing ChIP-seq data. Genome version mm9 and Ensembl annotation (release 67, excluding non-protein-coding transcripts) were used.

### Generation of bone marrow chimeras

Donor C57BL/6 and *Tet2*^*−/−*^ mice were injected with 150 μg g^−1^ of body weight of 5-FU purchased from Invivogen (San Diego, CA) by intraperitoneal injection (day −6). Six days post 5-FU injection (day 0), the donor mice were humanely euthanized; femurs and tibias were harvested. Bone marrow cells were flushed out and maintained overnight in 50 ng ml^−1^ SCF, 10 ng ml^−1^ IL-3 and 10 ng ml^−1^ IL-6, and transduced the following day (day 1). On day 2, transduction of donor cells was confirmed by flow cytometry and the recipient mice were irradiated to deliver 900 Rads 4–5 h before transfer of donor cells. The donor cells were extensively washed in PBS, re-suspended in PBS and 1.2 × 10^6^ cells were injected into each irradiated recipient mouse by retro-orbital injection. Transplantation was confirmed by bleeding 6 weeks post transfer.

## Additional information

**Accession codes:** RNA-seq and ChIP-seq data sets have been deposited in the NCBI Gene Expression Omnibus under GEO accession number GSE65555.

**How to cite this article:** Balasubramani, A. *et al.* Cancer-associated *ASXL1* mutations may act as gain-of-function mutations of the ASXL1–BAP1 complex. *Nat. Commun.* 6:7307 doi: 10.1038/ncomms8307 (2015).

## Supplementary Material

Supplementary Information Supplementary Figures 1-6 and Supplementary Table 1
