# Supplementary material for: Cancer-associated ASXL1 mutations may act as gain-of-function mutations of the ASXL1–BAP1 complex
Source: Nat Commun. 2015 Jun 22;6:7307. doi: 10.1038/ncomms8307 (PMC4557297; doi:10.1038/ncomms8307)
Supplement: Supplementary Information — Supplementary Figures 1-6 and Supplementary Table 1 [file ncomms8307-s1.pdf]

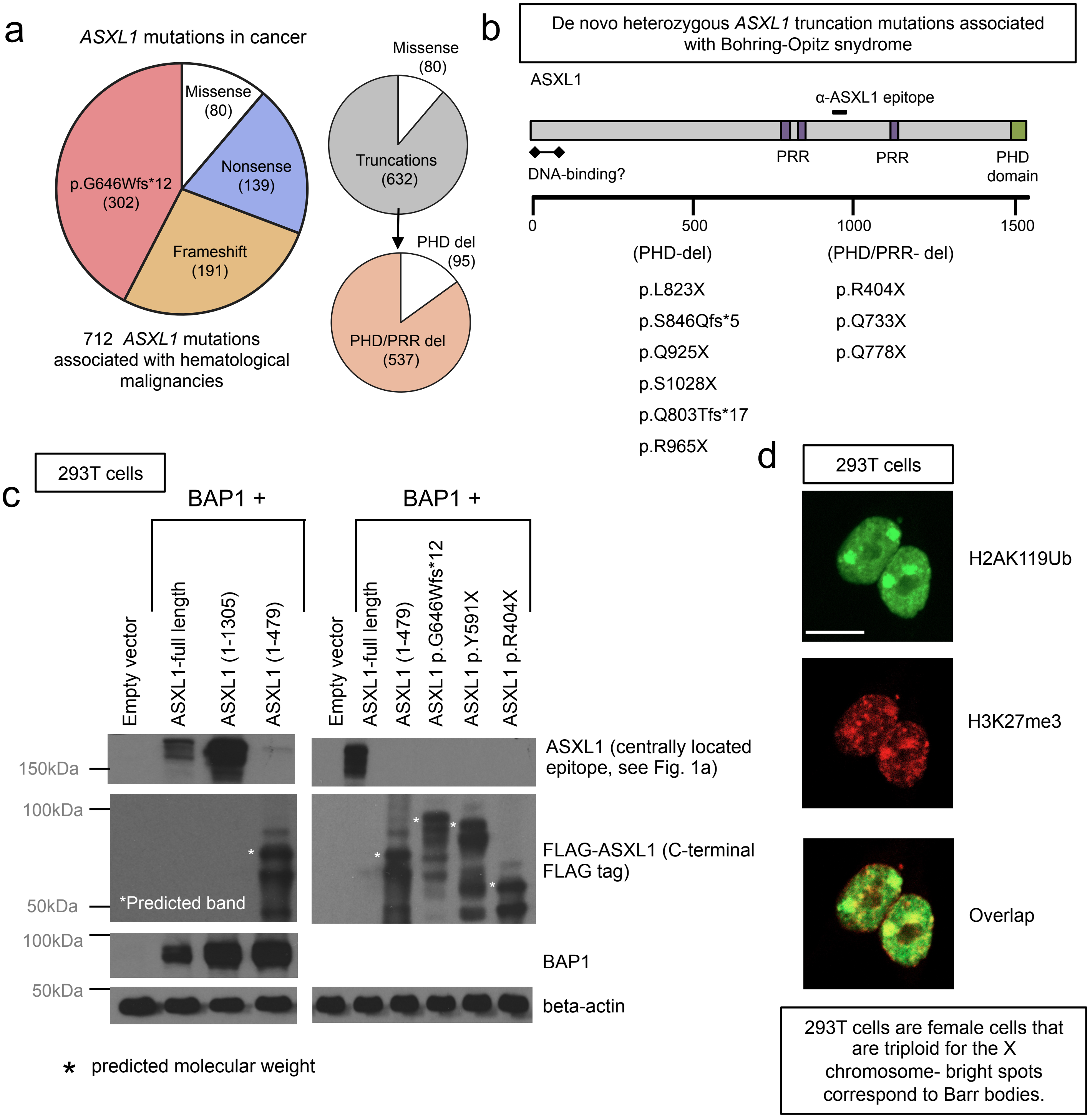

Supplementary Figure 1. ASXL1 mutations in hematological cancers and Bohring-Opitz syndrome (**a**) Summary of *ASXL1* mutations documented in patients with hematological malignancies. These mutations are invariably heterozygous and result in premature truncation of ASXL1 protein. Mutation data were obtained from the COSMIC database (**b**) *ASXL1* mutations documented in Bohring-Opitz syndrome patients have been tabulated here based on whether the truncation results in loss of just the PHD domain, or the PHD and all three PRR domains. (**c**) Expression of ASXL1 truncations was confirmed by Western blotting of nuclear lysates of transfected HEK293T cells. Anti-ASXL1 antibody was used to detect full length ASXL1 and ASXL1 (1-1305). All other ASXL1 truncations were tagged with a C-terminal 3XFLAG tag and these proteins were detected using anti-FLAG antibody. (**d**) HEK293T cells were fixed, permeabilized and co-stained with anti-H3K27me3 and H2AK119Ub antibodies. HEK293T cells are female and mostly triploid for the X chromosome; the two bright spots that co-stain with anti-H3K27me3 and H2AK119Ub antibodies correspond to the two inactive X chromosomes (Barr bodies). Scale bar 10μm.

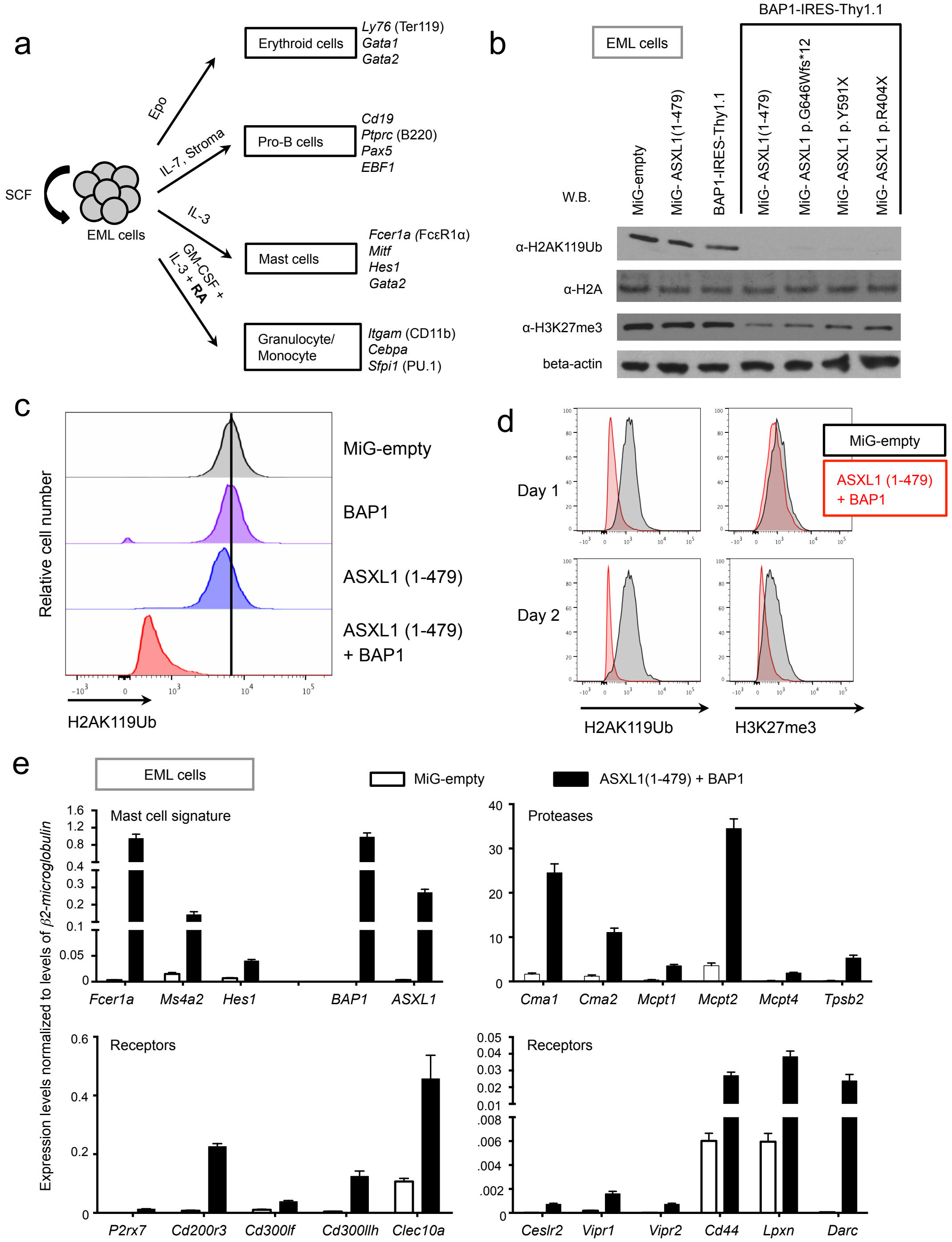

Supplementary Figure 2. Expression of ASXL1(1-479)+BAP1 in EML cells leads to upregulation of multiple mast cell associated genes. (**a**) Schematic overview of lineage potential of EML cells. When EML cells are maintained in SCF-containing media, they remain multipotent. EML cells can be induced to differentiate into distinct hematopoietic lineages by removing SCF and supplementing the media with cytokines and other co-factors as indicated. Genes characteristically expressed in the different lineages are listed to the right. (**b**) EML cells were transduced with the indicated retroviral constructs, FACS-sorted and expanded for 16 days in liquid culture in media supplemented with 100 ng/ml SCF. On day 16, nuclear lysates and acid-extracted histones prepared from these cells were subject to Western blotting with the indicated antibodies. (**c**) EML cells were transduced with MiG-empty, ASXL1(1-479), BAP1 or ASXL1(1-479) + BAP1. Cells were sorted based on reporter expression, expanded and subjected to intracellular staining with anti-H2AK119Ub antibody. (**d**) EML cells were transduced with MiG-empty, or ASXL1(1-479) + BAP1 and cells were subjected to intracellular staining with anti-H2AK119Ub and anti-H3K27me3 on day 1 and day 2 post-transduction. FACS plots are gated on transduced cells identified based on GFP/Thy1.1 expression. (**e**) EML cells transduced with either MiG-empty or ASXL1(1-479) + BAP1 were purified by FACS-sorting and expanded for 16 days in liquid culture. Upregulation of 20 mast cell-associated genes in ASXL1(1-479) + BAP1 transduced cells was evaluated by qPCR. Data represent mean ± SEM from four independent experiments.

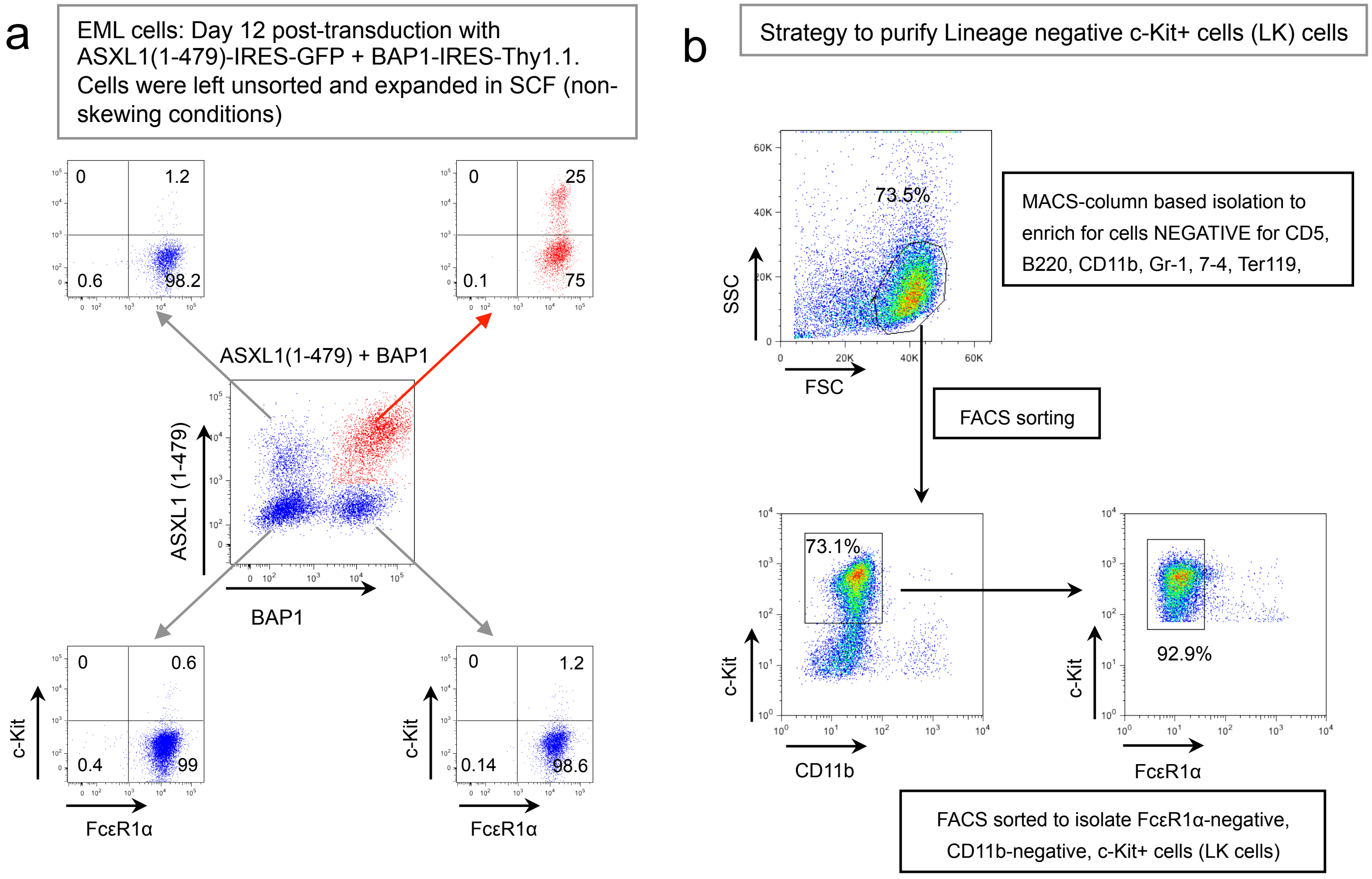

Supplementary Figure 3. ASXL1+BAP1 dependent skewing to the mast cell lineage is cell-intrinsic and contingent upon co-expression of both proteins. (**a**) EML cells transduced with ASXL1(1-479) + BAP1 were left unsorted and expanded in 100 ng/ml SCF. Only doubly-transduced cells undergo skewing to the mast cell lineage, indicating a cell-intrinsic effect. (**b**) To obtain purified LK cells, bone marrow cells were subject to lineage-depletion as indicated in experimental procedures. Subsequently, lineage-depleted cells were stained with fluorochrome-conjugated antibodies against c-Kit, CD11b and FcεR1α and LK cells were FACS-sorted by gating on c-Kit^{hi}, CD11b-negative and FcεR1α- negative cells.

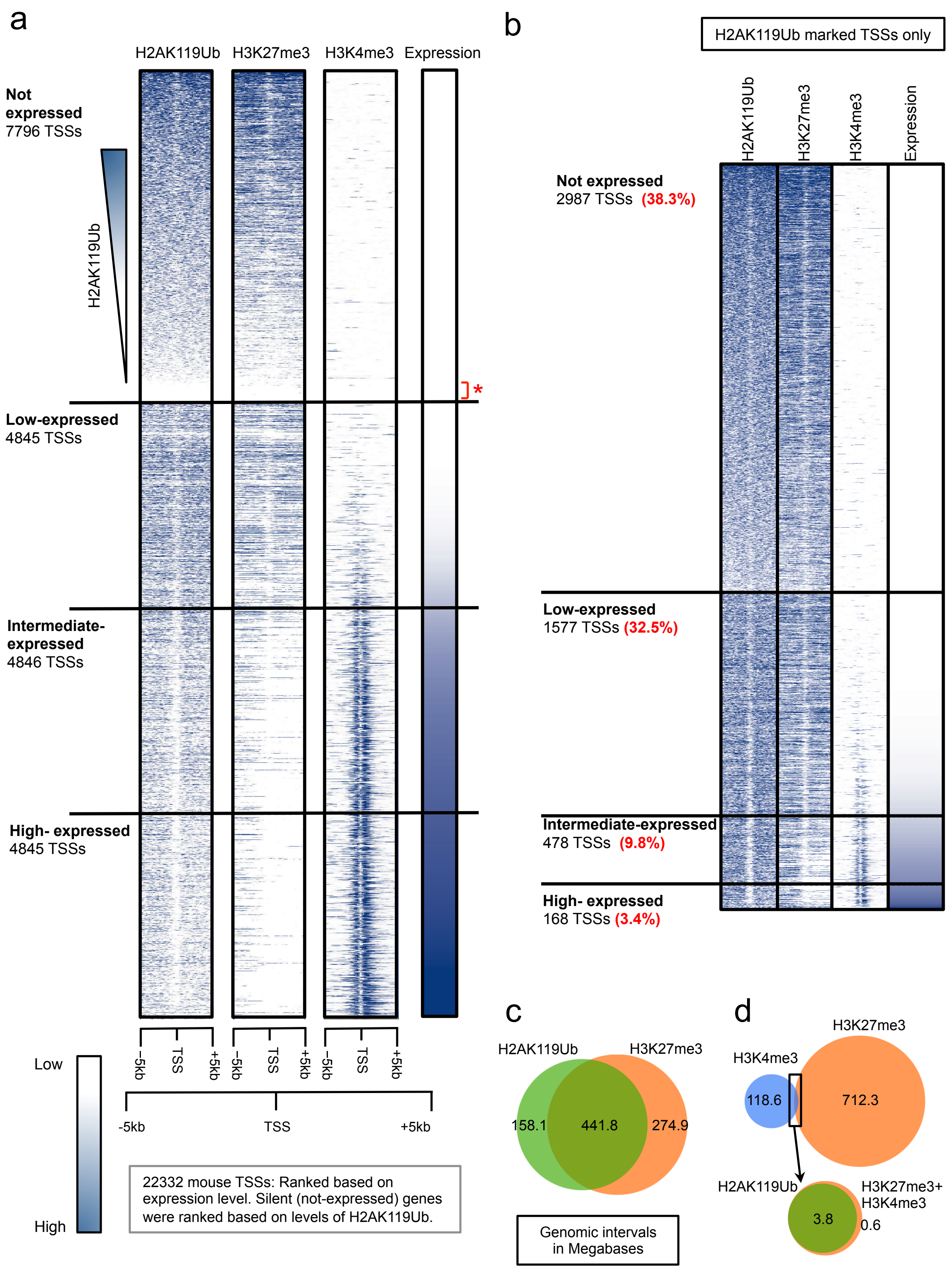

Supplementary Figure 4. Genome-wide distribution of H2AK119Ub closely overlaps with that of H3K27me3. (**a**) Input-corrected distribution of H2AK119Ub, H3K27me3 and H3K4me3 reads that were mapped to ±5kb around TSSs of all annotated protein-coding genes. As in Fig. 4a, TSSs were ranked based on expression levels and non-expressed genes were ordered based on levels of H2AK119Ub. (**b**) Same as in Supplementary Fig. 4a, but shown here is the distribution of H2AK119Ub, H3K4me3 and H3K27me3 marks restricted to TSSs ±5kb that are marked by H2AK119Ub. (c, d) Genomic intervals marked by H3K4me3, H2AK119Ub and H3K27me3 marks in EML cells were identified using SICER. BED-Intersect tool was used to identify overlapping regions. (**c**) Genome-wide overlap between H2AK119Ub and H3K27me3 is shown in the form of a Venn diagram. (**d**) Shown on top is the genome-wide overlap between H3K4me3 and H3K27me3 marks; overlap between H2AK119Ub and H3K27me3 in regions marked by both H3K4me3 and H3K27me3 marks is shown below.

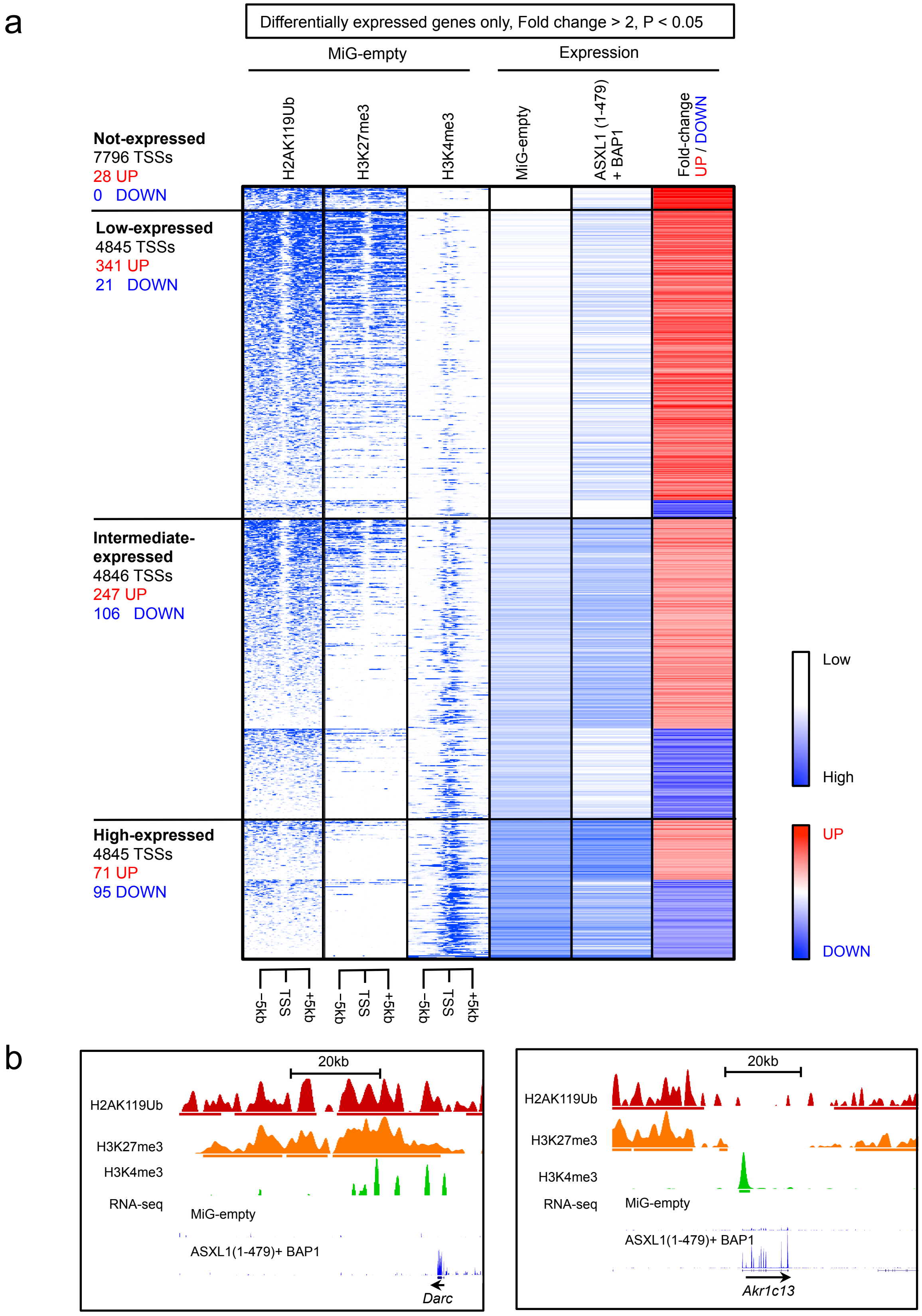

Supplementary Figure 5. Genes differentially expressed upon transduction of ASXL1(1-479) + BAP1 in EML cells. (**a**) Input-corrected distribution of H2AK119Ub, H3K4me3 and H3K27me3 marks ±5kb around mouse TSSs of differentially expressed genes (> 2-fold change, p-value<0.05, as determined by DESeq) is shown alongside gene expression levels in EML cells transduced with MiG-empty and ASXL1(1-479) + BAP1. Shown in the last column is the fold-change in gene expression (arcsinh-transformed). Genes within each sub-class (same as in **Fig. 4a**) were arranged in descending order of H2AK119Ub levels. (**b**) UCSC browser tracks of two genes upregulated in ASXL1(1-479)+BAP1 transduced EML cells, Akr1c13 and Darc showing H2AK119Ub, H3K27me3, H3K4me3 marks from MiG-empty transduced EML cells and RNA-seq tracks from both MiG-empty and ASXL1(1-479) + BAP1 of two genes. The bold lines below the profiles of histone marks represent SICER peak calls. Scale bar, 20kb.

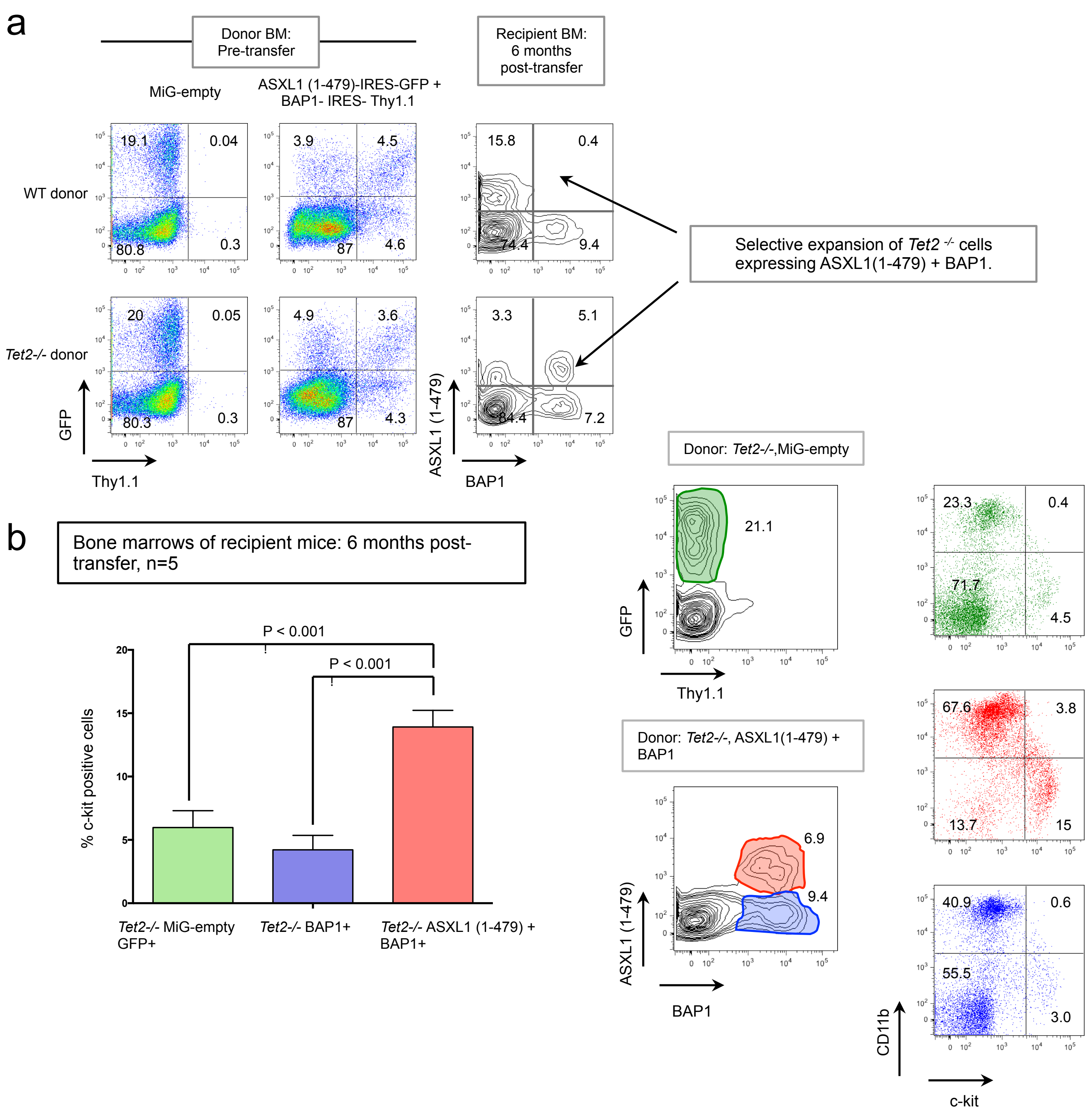

Supplementary Figure 6. *Tet2*$^{-/-}$ donor cells doubly-transduced with ASXL1(1-479)+BAP1 give rise to c-Kit+ precursor cells in the bone marrow of recipient mice. (**a**) Bone marrow cells harvested from 5-FU treated WT and *Tet2*$^{-/-}$ mice were cultured overnight in media supplemented with 50 ng/ml SCF, 10 ng/ml IL-3 and 10 ng/ml IL-6 and transduced the following day (day 1) with the indicated retroviruses. Transduction was confirmed by flow cytometry. Bulk (unsorted) transduced cells were transplanted into lethally irradiated recipient mice on day 2. Shown to the right are representative FACS plots of bone marrow cells 6 months post-transfer from mice that received WT and *Tet2*$^{-/-}$ bone marrow cells transduced with ASXL1(1-479)+ BAP1. (**b**) Transduced cells were identified based on expression of GFP and Thy1.1 reporters. Percentages of c-kit positive cells that were transduced with either MiG-empty, BAP1 or ASXL1(1-479) + BAP1 were determined by flow cytometry. Data shown are from recipients that received cells from TET2-deficient donors; percentages represent mean ± SD from 5 recipients. Statistical significance was determined by two-tailed t-test. Representative FACS-plots are shown to the right.

Supplementary Table 1
List of genes that are differentially expressed in EML cells transduced with ASXL1(1-479) + BAP1 as compared to EML cells transduced with MiG-empty. The genes are split into 4 groups, that correspond to the 4 subsets of differentially expressed genes indicated in **Figure 2d**.

375 genes upregulated >4-fold in EML cells transduced with ASXL1(1-479)+BAP1 (P<0.05, DE-seq)

ENSEML ID	*Gene name*	*MiG empty expression*	*ASXL1(1-479)+BAP1*	*log2 fold-change*	*p-value*
ENSMUSG00000042363	*1110067D22Rik*	0.0000	1053.4595	∞	2.09E-13
ENSMUSG00000027002	*Nckap1*	0.0000	150.0718	∞	1.74E-12
ENSMUSG00000025265	*Fgd1*	0.0000	183.6584	∞	1.16E-09
ENSMUSG00000040616	*Tmem51*	0.0000	404.4230	∞	1.86E-08
ENSMUSG00000010830	*Kdelr3*	0.0000	251.8084	∞	4.46E-05
ENSMUSG00000027398	*Il1b*	0.0000	164.0148	∞	0.000257512
ENSMUSG00000040836	*Gpr161*	0.0000	69.5958	∞	0.000834367
ENSMUSG00000002076	*Hsf2bp*	0.0000	146.3505	∞	0.001377499
ENSMUSG00000037217	*Syn1*	0.0000	50.1747	∞	0.001562363
ENSMUSG00000026580	*Selp*	0.0000	38.1773	∞	0.004047954
ENSMUSG00000052922	*Bpi*	0.0000	57.7958	∞	0.005229521
ENSMUSG00000026411	*Tmem9*	0.0000	130.6686	∞	0.011338513
ENSMUSG00000032387	*Rbpms2*	0.0000	117.0673	∞	0.016746652
ENSMUSG00000037716	*Ccdc33*	0.0000	29.1789	∞	0.024782342
ENSMUSG00000036098	*Gm98*	0.0000	17.7538	∞	0.030168277
ENSMUSG00000020080	*Hkdc1*	0.0000	18.7721	∞	0.041909653
ENSMUSG00000032011	*Thy1*	10.1824	12001.9150	10.2030	1.16E-44
ENSMUSG00000026825	*Dnm1*	0.4794	206.5354	8.7509	2.76E-10
ENSMUSG00000019852	*D10Bwg1379e*	0.3041	103.9870	8.4178	4.36E-14
ENSMUSG00000041707	*1810011H11Rik*	27.7818	6998.2193	7.9767	1.88E-25
ENSMUSG00000050425	*Mrgprb2*	13.6648	3334.0587	7.9307	2.34E-31
ENSMUSG00000050947	*Amigo1*	1.3753	323.6309	7.8784	1.96E-10
ENSMUSG00000026581	*Sell*	10.8662	2529.1885	7.8627	6.64E-29
ENSMUSG00000061410	*Zcchc14*	1.3385	310.1741	7.8563	1.09E-13
ENSMUSG00000037482	*Erv3*	51.1358	8621.9196	7.3975	2.57E-33
ENSMUSG00000030084	*Plxna1*	0.3484	55.6581	7.3198	1.66E-07
ENSMUSG00000027624	*Epb4.1l1*	7.7656	1220.8608	7.2966	3.80E-27
ENSMUSG00000024486	*Hbegf*	1.5861	238.0225	7.2295	0.000327969
ENSMUSG00000037872	*Darc*	0.9877	139.1543	7.1383	0.000543456
ENSMUSG00000004540	*Psg17*	0.6945	92.1751	7.0522	0.000853152
ENSMUSG00000074109	*Mrgprx2*	20.6193	2553.7002	6.9525	1.61E-25
ENSMUSG00000032327	*Stra6*	1.0850	112.9841	6.7022	0.004190786
ENSMUSG00000031443	*F7*	0.9703	97.0758	6.6445	0.00526792
ENSMUSG00000028238	*Atp6v0d2*	3.4187	301.8928	6.4644	0.000182737
ENSMUSG00000028763	*Hspg2*	0.1744	15.3088	6.4561	0.000192497
ENSMUSG00000022098	*Bmp1*	12.4592	1085.9014	6.4455	3.02E-21
ENSMUSG00000005339	*Fcer1a*	128.0045	10688.4860	6.3837	7.80E-31
ENSMUSG00000041115	*Iqsec2*	0.5038	41.1477	6.3517	0.000360038
ENSMUSG00000028909	*Ptpru*	2.3839	194.3408	6.3491	3.38E-10
ENSMUSG00000015709	*Arnt2*	0.9267	74.2649	6.3244	0.000421244

ENSMUSG00000030737	*Slco2b1*	7.9294	627.3565	6.3059	1.67E-12
ENSMUSG00000004864	*Mapk13*	1.8028	142.6312	6.3059	0.00046777
ENSMUSG00000040711	*Sh3pxd2b*	2.9067	222.1800	6.2562	1.55E-10
ENSMUSG00000003352	*Cacnb3*	4.7763	344.1078	6.1708	3.76E-09
ENSMUSG00000068740	*Celsr2*	1.4355	103.1659	6.1673	3.15E-07
ENSMUSG00000006345	*Ggt1*	1.6441	115.8078	6.1383	0.027783569
ENSMUSG00000029468	*P2rx7*	82.7380	5798.3919	6.1310	3.97E-29
ENSMUSG00000015647	*Lama5*	3.9857	271.9996	6.0926	1.35E-20
ENSMUSG00000041757	*Plekha6*	0.3088	20.8082	6.0742	0.032995516
ENSMUSG00000025743	*Sdc3*	12.0481	747.5363	5.9553	1.74E-09
ENSMUSG00000035385	*Ccl2*	71.3316	4387.4211	5.9427	5.94E-19
ENSMUSG00000070547	*Mrgprb1*	232.8211	13349.5414	5.8414	1.33E-29
ENSMUSG00000059668	*Krt4*	2.5136	136.6420	5.7645	8.77E-05
ENSMUSG00000025780	*Itih5*	11.4359	617.3373	5.7544	1.66E-17
ENSMUSG00000016128	*Stard13*	2.1230	112.1592	5.7233	0.000116176
ENSMUSG00000021338	*Lrrc16a*	8.1468	427.3934	5.7132	1.36E-12
ENSMUSG00000037202	*Prf1*	5.3595	276.6086	5.6896	4.87E-08
ENSMUSG00000051079	*Rgs13*	27.1444	1359.9646	5.6468	5.97E-10
ENSMUSG00000033825	*Tpsb2*	1340.4977	66497.4603	5.6325	2.44E-30
ENSMUSG00000021211	*Akr1c12*	48.0053	2327.3177	5.5993	9.08E-19
ENSMUSG00000023830	*Igf2r*	2.3272	108.0996	5.5376	1.63E-05
ENSMUSG00000001211	*Agpat3*	78.7779	3398.1488	5.4308	2.79E-19
ENSMUSG00000074676	*Foxs1*	2.0055	85.9899	5.4221	0.01979223
ENSMUSG00000009545	*Kcnq1*	1.1889	50.3674	5.4048	0.020861469
ENSMUSG00000034871	*Fam151a*	42.3238	1784.7852	5.3981	2.38E-20
ENSMUSG00000042351	*Grap2*	20.4912	858.7194	5.3891	4.78E-11
ENSMUSG00000029304	*Spp1*	49.4435	2061.8419	5.3820	2.17E-14
ENSMUSG00000029413	*Naaa*	58.0123	2323.6973	5.3239	1.94E-17
ENSMUSG00000032997	*Chpf*	18.5663	738.2247	5.3133	5.31E-15
ENSMUSG00000040289	*Hey1*	2.2067	86.7328	5.2966	0.028589861
ENSMUSG00000061436	*Hipk2*	36.1485	1412.6885	5.2884	1.35E-20
ENSMUSG00000032340	*Neo1*	7.4028	286.9464	5.2766	1.44E-13
ENSMUSG00000015981	*Stk32c*	15.3803	583.3048	5.2451	4.07E-10
ENSMUSG00000034586	*C630004H02Rik*	5.8923	219.8304	5.2214	8.18E-07
ENSMUSG00000050870	*Mrgprb8*	24.9931	913.4254	5.1917	3.84E-11
ENSMUSG00000019558	*Slc6a8*	27.0204	983.0058	5.1851	8.70E-15
ENSMUSG00000040046	*Tph1*	17.9264	652.0763	5.1849	1.89E-09
ENSMUSG00000038094	*Atp13a4*	2.5488	92.6955	5.1846	2.32E-05
ENSMUSG00000030257	*Srgap3*	6.9309	246.5811	5.1529	1.49E-09
ENSMUSG00000036172	*Cd200r3*	371.0136	13107.9795	5.1428	6.02E-24
ENSMUSG00000035168	*Tanc1*	6.0595	208.2622	5.1030	5.02E-11
ENSMUSG00000031442	*Mcf2l*	2.3554	78.5496	5.0595	0.000462513
ENSMUSG00000011171	*Vipr2*	5.8982	187.8659	4.9933	0.001911292
ENSMUSG00000031217	*Efnb1*	5.7375	179.6233	4.9684	0.000782459
ENSMUSG00000026883	*Dab2ip*	22.4560	690.2523	4.9420	5.14E-16
ENSMUSG00000025701	*Alox5*	12.8004	393.0712	4.9405	1.52E-09
ENSMUSG00000027901	*Dennd2d*	16.9365	518.6984	4.9367	2.70E-09
ENSMUSG00000004267	*Eno2*	16.6072	477.3146	4.8451	6.01E-06

ENSMUSG00000034591	*Slc41a2*	130.8728	3754.2760	4.8423	9.72E-16
ENSMUSG00000024424	*Ttc39c*	20.9561	589.8475	4.8149	1.17E-10
ENSMUSG00000041817	*Fam169a*	8.9299	251.2025	4.8141	3.39E-07
ENSMUSG00000056399	*Prss34*	8.2996	233.0020	4.8112	0.000332313
ENSMUSG00000020032	*Nuak1*	4.0364	112.0814	4.7953	0.004735268
ENSMUSG00000022225	*Cma1*	21082.4211	583286.5931	4.7901	9.03E-25
ENSMUSG00000032014	*Oaf*	92.4212	2543.8676	4.7827	3.46E-15
ENSMUSG00000027488	*Snta1*	2.6340	69.9133	4.7302	0.015073252
ENSMUSG00000051159	*Cited1*	11.8502	312.1149	4.7191	0.006497928
ENSMUSG00000017466	*Timp2*	203.3931	5344.8239	4.7158	1.53E-16
ENSMUSG00000067173	*Mrgpra4*	18.9724	477.7521	4.6543	1.79E-06
ENSMUSG00000032528	*Vipr1*	52.4691	1289.2083	4.6189	1.78E-13
ENSMUSG00000035638	*Muc20*	1.9637	47.6122	4.5997	0.022941475
ENSMUSG00000042066	*Tmcc2*	4.3866	105.7961	4.5920	0.001189396
ENSMUSG00000033200	*Tpsg1*	101.7628	2434.6333	4.5804	2.74E-12
ENSMUSG00000041235	*Chd7*	4.1437	96.1801	4.5368	3.41E-06
ENSMUSG00000029710	*Ephb4*	4.0552	92.4566	4.5109	0.000161375
ENSMUSG00000037185	*Krt80*	3.9216	89.0777	4.5055	0.030305674
ENSMUSG00000069607	*Cd300lh*	8.0794	181.4562	4.4892	0.007582865
ENSMUSG00000020331	*Hcn2*	2.2937	51.1240	4.4783	0.00791873
ENSMUSG00000024462	*Gabbr1*	27.7582	597.0190	4.4268	1.88E-09
ENSMUSG00000032198	*Dock6*	2.4658	52.8620	4.4221	0.000173205
ENSMUSG00000035373	*Ccl7*	248.2816	5225.5110	4.3955	7.43E-12
ENSMUSG00000021913	*Ogdhl*	46.1680	965.5408	4.3864	3.86E-15
ENSMUSG00000068289	*Cma2*	40.6522	839.5901	4.3683	1.00E-06
ENSMUSG00000066607	*6030419C18Rik*	5.6491	116.4937	4.3661	0.022790961
ENSMUSG00000028278	*Rragd*	68.1966	1395.0758	4.3545	6.56E-12
ENSMUSG00000040533	*Matn1*	9.2374	184.5630	4.3205	0.000212296
ENSMUSG00000063564	*Col23a1*	24.2038	482.3617	4.3168	5.07E-06
ENSMUSG00000071552	*Tigit*	19.1648	357.7560	4.2224	0.000403792
ENSMUSG00000061068	*Mcpt4*	5118.9459	95542.6325	4.2222	2.78E-20
ENSMUSG00000028943	*Espn*	6.5304	121.8179	4.2214	0.001572189
ENSMUSG00000052889	*Prkcb*	88.3683	1630.8435	4.2059	1.02E-14
ENSMUSG00000021638	*Ocln*	14.4399	257.5379	4.1567	0.000183959
ENSMUSG00000056214	*Pard6g*	24.7652	438.8251	4.1473	4.69E-06
ENSMUSG00000001930	*Vwf*	78.2684	1367.5103	4.1270	6.38E-16
ENSMUSG00000025432	*Avil*	17.1413	297.4842	4.1173	2.96E-05
ENSMUSG00000021662	*Rgnef*	2.1009	36.0314	4.1002	0.004474164
ENSMUSG00000022514	*Il1rap*	21.0673	360.8398	4.0983	8.41E-07
ENSMUSG00000022528	*Hes1*	31.5870	541.0197	4.0983	1.00E-05
ENSMUSG00000039145	*Camk1d*	154.8979	2652.3656	4.0979	1.00E-13
ENSMUSG00000006576	*Slc4a3*	7.0647	120.0300	4.0866	0.000917629
ENSMUSG00000056501	*Cebpb*	18.9474	319.7265	4.0768	0.000315927
ENSMUSG00000035275	*Raver2*	3.4973	58.3141	4.0595	0.032569823
ENSMUSG00000029769	*Ccdc136*	3.9457	65.4566	4.0522	0.002433963
ENSMUSG00000026950	*Neb*	2.6713	44.0877	4.0447	3.92E-05
ENSMUSG00000006219	*Fblim1*	168.0228	2754.2181	4.0349	2.39E-10
ENSMUSG00000048264	*Dip2c*	12.5662	205.7140	4.0330	2.58E-08

ENSMUSG00000000732	*Icosl*	42.4098	669.5600	3.9807	5.94E-07
ENSMUSG00000028434	*Epb4.1l4b*	30.1562	475.3286	3.9784	1.51E-07
ENSMUSG00000037010	*Apln*	269.9251	4242.7263	3.9744	1.69E-08
ENSMUSG00000069919	*Hba-a1*	14.0387	217.3633	3.9526	0.043721862
ENSMUSG00000041592	*Sdk2*	2.4435	37.1409	3.9260	0.013700601
ENSMUSG00000036687	*Tmem184a*	15.4846	233.1649	3.9124	0.004725453
ENSMUSG00000027861	*Casq2*	6.6944	99.9489	3.9002	0.022159075
ENSMUSG00000027048	*Abcb11*	2.2477	33.3989	3.8933	0.015397945
ENSMUSG00000047798	*Cd300lf*	216.0556	3192.7830	3.8853	7.48E-13
ENSMUSG00000028457	*Atp8b5*	281.1317	4113.9536	3.8712	6.13E-16
ENSMUSG00000028108	*Ecm1*	46.4154	674.1459	3.8604	1.74E-06
ENSMUSG00000028965	*Tnfrsf9*	22.3667	322.7131	3.8508	0.002321064
ENSMUSG00000001473	*Tubb6*	22.8796	329.2592	3.8471	3.11E-05
ENSMUSG00000001864	*Aif1l*	228.7365	3264.6539	3.8352	8.11E-10
ENSMUSG00000032281	*Acsbg1*	86.3714	1227.8550	3.8294	5.79E-08
ENSMUSG00000036526	*Card11*	4.2882	60.7046	3.8233	0.002667053
ENSMUSG00000028716	*Pdzk1ip1*	447.6753	6329.0872	3.8215	4.84E-12
ENSMUSG00000030762	*Aqp8*	88.8495	1252.0103	3.8167	1.60E-07
ENSMUSG00000072812	*Ahnak2*	130.1820	1831.3685	3.8143	4.35E-15
ENSMUSG00000030688	*Stard10*	57.0995	799.0163	3.8067	5.32E-06
ENSMUSG00000044461	*Shisa2*	10.0650	140.4259	3.8024	0.020994379
ENSMUSG00000022226	*Mcpt2*	52022.7823	722240.3620	3.7953	1.38E-17
ENSMUSG00000040552	*C3ar1*	76.7712	1060.5119	3.7881	8.54E-10
ENSMUSG00000012889	*Podnl1*	20.0660	272.9294	3.7657	3.17E-05
ENSMUSG00000032220	*Myo1e*	241.3442	3246.6588	3.7498	2.57E-14
ENSMUSG00000024696	*Lpxn*	208.5432	2798.1801	3.7461	1.66E-12
ENSMUSG00000021477	*Ctsl*	80.2819	1074.4104	3.7423	2.47E-07
ENSMUSG00000048416	*Mlf1*	57.6889	766.8346	3.7325	3.12E-06
ENSMUSG00000066877	*Nck2*	280.4222	3725.1160	3.7316	2.74E-13
ENSMUSG00000045312	*Lhfpl2*	92.4793	1213.2609	3.7136	4.45E-06
ENSMUSG00000005087	*Cd44*	593.1513	7779.4005	3.7132	1.29E-15
ENSMUSG00000000318	*Clec10a*	2727.7582	35464.2894	3.7006	5.99E-16
ENSMUSG00000000127	*Fer*	71.7299	919.3491	3.6800	3.33E-10
ENSMUSG00000027204	*Fbn1*	9.4161	120.3923	3.6765	6.07E-08
ENSMUSG00000030930	*Chst15*	18.2302	232.9340	3.6755	0.000121494
ENSMUSG00000069917	*Hba-a2*	72.5331	910.0597	3.6493	0.000147668
ENSMUSG00000028359	*Orm3*	27.2255	340.0636	3.6428	0.003685222
ENSMUSG00000071361	*Mcpt9*	52.3026	644.8368	3.6240	3.98E-05
ENSMUSG00000014599	*Csf1*	518.5463	6176.3051	3.5742	2.07E-13
ENSMUSG00000024173	*Tpsab1*	74.4955	881.0959	3.5641	8.56E-07
ENSMUSG00000001313	*Rnd2*	24.7068	290.7999	3.5570	0.005492576
ENSMUSG00000037902	*Sirpa*	409.2649	4797.9453	3.5513	5.76E-13
ENSMUSG00000037949	*Ano10*	134.4552	1560.2215	3.5366	2.79E-11
ENSMUSG00000050439	*Enthd1*	4.8045	55.5873	3.5323	0.046804109
ENSMUSG00000026012	*Cd28*	148.3075	1694.7832	3.5144	9.53E-08
ENSMUSG00000043987	*Cep164*	41.9640	473.6349	3.4965	5.20E-09
ENSMUSG00000039470	*Zdhhc2*	121.6876	1364.6588	3.4873	9.45E-09
ENSMUSG00000054293	*A630033H20Rik*	82.9750	921.6086	3.4734	2.60E-05

ENSMUSG00000032323	*Cyp11a1*	3016.2166	32976.3326	3.4506	6.50E-15
ENSMUSG00000028602	*Tnfrsf8*	37.0981	404.4230	3.4464	1.50E-05
ENSMUSG00000031955	*Bcar1*	18.1255	195.4457	3.4307	4.92E-05
ENSMUSG00000024130	*Abca3*	4.0755	43.4774	3.4152	0.005436748
ENSMUSG00000040219	*Ttc12*	19.6820	208.5825	3.4057	0.000139246
ENSMUSG00000074796	*Slc4a11*	19.2866	204.1108	3.4037	0.000920627
ENSMUSG00000018648	*Dusp14*	22.7523	236.1427	3.3756	0.034351499
ENSMUSG00000064325	*Hhip*	21.2084	218.4348	3.3645	0.000125264
ENSMUSG00000053617	*Sh3pxd2a*	38.0779	391.4806	3.3619	7.63E-08
ENSMUSG00000004562	*Arhgef40*	17.0528	174.9911	3.3592	3.54E-06
ENSMUSG00000024680	*Ms4a2*	1562.6311	15992.2703	3.3553	6.86E-13
ENSMUSG00000073424	*Cyp4f15*	8.0845	81.8971	3.3406	0.030663995
ENSMUSG00000034684	*Sema3f*	16.2918	165.0049	3.3403	0.000396108
ENSMUSG00000031996	*Aplp2*	37.5572	377.1252	3.3279	2.43E-05
ENSMUSG00000055538	*Zcchc24*	135.5226	1347.1749	3.3133	8.99E-07
ENSMUSG00000032194	*Kank2*	28.2129	279.6549	3.3092	2.57E-05
ENSMUSG00000020838	*Slc6a4*	412.3081	4070.2504	3.3033	7.55E-12
ENSMUSG00000026308	*Klhl30*	61.3255	602.2869	3.2959	5.71E-07
ENSMUSG00000031444	*F10*	990.0437	9674.3188	3.2886	1.12E-12
ENSMUSG00000035183	*Slc24a5*	9.2190	89.4083	3.2777	0.030331632
ENSMUSG00000038390	*Gpr162*	145.8615	1396.3419	3.2590	6.26E-09
ENSMUSG00000029869	*Ephb6*	52.7487	504.8400	3.2586	2.03E-06
ENSMUSG00000029314	*Agpat9*	22.3358	212.8185	3.2522	0.015582379
ENSMUSG00000051341	*Zfp52*	19.1383	179.5613	3.2299	0.000485112
ENSMUSG00000046269	*Usp27x*	18.8304	174.5484	3.2125	0.005730533
ENSMUSG00000020122	*Egfr*	14.9732	137.5708	3.1997	0.002900304
ENSMUSG00000030761	*Myo7a*	57.0430	519.2118	3.1862	3.97E-09
ENSMUSG00000021213	*Akr1c13*	952.9555	8546.0107	3.1648	1.01E-11
ENSMUSG00000019832	*Rab32*	122.7911	1091.9366	3.1526	1.57E-05
ENSMUSG00000041012	*Cmtm8*	83.9074	745.6185	3.1516	0.000638772
ENSMUSG00000028339	*Col15a1*	85.6543	757.6526	3.1449	1.62E-07
ENSMUSG00000022227	*Mcpt1*	61.6902	544.8939	3.1429	0.000538766
ENSMUSG00000022372	*Sla*	489.6536	4318.0502	3.1405	4.34E-10
ENSMUSG00000054871	*Tmem158*	72.6721	639.4371	3.1373	9.87E-05
ENSMUSG00000020182	*Ddc*	93.0162	817.9673	3.1365	2.20E-06
ENSMUSG00000047953	*Gp5*	39.5657	345.1096	3.1247	6.94E-05
ENSMUSG00000027777	*Schip1*	27.3413	235.2572	3.1051	0.006093514
ENSMUSG00000047793	*Sned1*	19.0332	162.9427	3.0978	0.000235805
ENSMUSG00000017692	*Rhbdl3*	21.2317	180.9016	3.0909	0.008524347
ENSMUSG00000029570	*Lfng*	204.2291	1704.4614	3.0611	4.23E-08
ENSMUSG00000044468	*Fam46c*	267.4706	2221.4605	3.0541	7.20E-09
ENSMUSG00000027219	*Slc28a2*	7.4979	62.2558	3.0536	0.048941495
ENSMUSG00000026473	*Glul*	110.4539	910.9751	3.0440	7.00E-05
ENSMUSG00000002233	*Rhoc*	166.4959	1308.7742	2.9747	4.67E-05
ENSMUSG00000063450	*Syne2*	30.9754	238.5039	2.9448	9.85E-07
ENSMUSG00000043505	*Gimap5*	36.4185	278.8390	2.9367	0.006446497
ENSMUSG00000020027	*Socs2*	178.1011	1355.7795	2.9284	0.000124683
ENSMUSG00000045045	*Lrfn4*	12.4494	94.2599	2.9206	0.021540707

ENSMUSG00000035914	*Cd276*	93.9613	707.6932	2.9130	7.88E-05
ENSMUSG00000049791	*Fzd4*	49.1484	366.2710	2.8977	0.000169569
ENSMUSG00000049047	*Armcx3*	73.1196	536.7372	2.8759	0.000154856
ENSMUSG00000037922	*Bank1*	21.8689	160.1046	2.8721	0.007810503
ENSMUSG00000022219	*Cideb*	33.9743	246.4344	2.8587	0.048244122
ENSMUSG00000025085	*Ablim1*	28.9020	207.0931	2.8410	0.001812513
ENSMUSG00000074217	*2210011C24Rik*	63.3424	452.6508	2.8372	0.02869232
ENSMUSG00000015243	*Abca1*	18.3850	131.0829	2.8339	2.34E-05
ENSMUSG00000039168	*Dap*	520.7379	3658.5045	2.8126	7.03E-06
ENSMUSG00000072214	*5-Sep*	238.9168	1669.9572	2.8052	8.72E-07
ENSMUSG00000052155	*Acvr2a*	41.8013	292.1505	2.8051	0.000913953
ENSMUSG00000037346	*Hrh4*	199.4409	1389.5923	2.8006	8.22E-06
ENSMUSG00000047037	*Nipa1*	60.2619	414.9966	2.7838	0.001603209
ENSMUSG00000027360	*Hdc*	527.2556	3625.8745	2.7818	1.60E-09
ENSMUSG00000026418	*Tnni1*	67.6881	461.3493	2.7689	0.013388858
ENSMUSG00000041570	*Camsap2*	28.8857	196.1328	2.7634	3.89E-05
ENSMUSG00000016529	*Il10*	79.6970	541.1405	2.7634	0.006225716
ENSMUSG00000052187	*Hbb-y*	641.8628	4343.9518	2.7587	5.05E-07
ENSMUSG00000004730	*Emr1*	86.1792	581.1489	2.7535	2.52E-05
ENSMUSG00000035314	*Gdpd5*	47.8286	321.2579	2.7478	0.000322347
ENSMUSG00000038453	*Srcin1*	22.4501	146.4934	2.7060	0.000965861
ENSMUSG00000022994	*Adcy6*	130.2122	844.8678	2.6979	1.00E-07
ENSMUSG00000090176	*Cd200r2*	80.8210	522.0979	2.6915	0.002391074
ENSMUSG00000064262	*Gimap8*	157.7615	1018.2136	2.6902	5.41E-07
ENSMUSG00000032289	*Thsd4*	19.1319	122.9035	2.6835	0.010547224
ENSMUSG00000004791	*Pgf*	56.3768	355.9782	2.6586	0.037764013
ENSMUSG00000022438	*Parvb*	637.2205	3969.5525	2.6391	3.81E-08
ENSMUSG00000025006	*Sorbs1*	32.0443	199.0709	2.6351	0.000975147
ENSMUSG00000040964	*Arhgef10l*	230.0569	1428.8427	2.6348	5.59E-08
ENSMUSG00000005611	*Mrvi1*	384.8721	2381.9464	2.6297	5.07E-08
ENSMUSG00000089942	*Pira2*	120.9261	746.5271	2.6261	6.25E-06
ENSMUSG00000022957	*Itsn1*	21.7213	133.8844	2.6238	0.005827093
ENSMUSG00000018500	*Adora2b*	54.6535	336.7937	2.6235	0.00511442
ENSMUSG00000046245	*Pilra*	889.2065	5475.5294	2.6224	5.07E-08
ENSMUSG00000036596	*Cpz*	288.0153	1773.0675	2.6220	1.16E-06
ENSMUSG00000034413	*Neurl1b*	73.1116	441.6563	2.5948	0.000220473
ENSMUSG00000029005	*2610109H07Rik*	35.5878	214.0555	2.5885	0.021075468
ENSMUSG00000039395	*Mreg*	141.8298	846.7057	2.5777	0.000865094
ENSMUSG00000019726	*Lyst*	89.6011	528.2854	2.5597	6.56E-08
ENSMUSG00000021054	*Sgpp1*	182.2602	1073.4462	2.5582	2.16E-05
ENSMUSG00000040147	*Maob*	25.3776	148.9665	2.5534	0.019352818
ENSMUSG00000020821	*Kif1c*	223.7276	1308.7017	2.5483	9.64E-08
ENSMUSG00000031520	*Vegfc*	157.5189	918.7328	2.5441	0.000149628
ENSMUSG00000051124	*Gimap9*	369.1284	2130.6718	2.5291	1.43E-05
ENSMUSG00000023186	*Vwa5a*	4426.3399	25519.5913	2.5274	3.09E-09
ENSMUSG00000090019	*Gimap1*	200.7085	1155.1456	2.5249	0.003757205
ENSMUSG00000035112	*Wnk4*	16.9560	97.1274	2.5181	0.029218144
ENSMUSG00000022415	*Syngr1*	714.7466	4092.1959	2.5174	2.45E-06

ENSMUSG00000069171	*Nr2f1*	54.1277	309.4413	2.5152	0.011697548
ENSMUSG00000074417	*Gm14548*	89.1904	508.7152	2.5119	0.000133532
ENSMUSG00000063455	*D630045J12Rik*	94.6606	538.6131	2.5084	4.21E-06
ENSMUSG00000037306	*Man1c1*	273.1145	1550.8251	2.5055	1.25E-06
ENSMUSG00000031788	*Kifc3*	201.9230	1141.0378	2.4985	9.97E-07
ENSMUSG00000003070	*Efna2*	59.9834	338.4052	2.4961	0.027455164
ENSMUSG00000023951	*Vegfa*	226.2755	1271.8972	2.4908	0.000122393
ENSMUSG00000035969	*Rusc2*	283.3792	1570.3507	2.4703	2.15E-06
ENSMUSG00000030616	*Sytl2*	11.3371	62.4978	2.4628	0.037694089
ENSMUSG00000021879	*Dnahc12*	77.2412	423.2518	2.4541	2.30E-05
ENSMUSG00000016028	*Celsr1*	10.2966	56.3927	2.4533	0.004292809
ENSMUSG00000036959	*Bcorl1*	50.3660	275.7218	2.4527	3.68E-05
ENSMUSG00000054074	*2810030E01Rik*	21.7269	118.2951	2.4448	0.045696918
ENSMUSG00000048458	*6530418L21Rik*	140.8215	766.3362	2.4441	0.000605939
ENSMUSG00000026791	*Slc2a8*	298.2734	1620.5315	2.4418	2.14E-05
ENSMUSG00000022537	*Tmem44*	644.2000	3469.9719	2.4293	1.16E-06
ENSMUSG00000037139	*Myom3*	268.6954	1443.6023	2.4256	1.20E-07
ENSMUSG00000031391	*L1cam*	352.7734	1890.3132	2.4218	6.14E-07
ENSMUSG00000020021	*Fgd6*	44.3337	236.8871	2.4177	0.00012169
ENSMUSG00000020520	*Galnt10*	609.6816	3247.2948	2.4131	6.60E-07
ENSMUSG00000026640	*Plxna2*	118.2724	622.9084	2.3969	8.03E-07
ENSMUSG00000031565	*Fgfr1*	245.6873	1293.7091	2.3966	5.39E-06
ENSMUSG00000049858	*Suox*	124.4710	650.7645	2.3863	0.000106315
ENSMUSG00000020092	*X99384*	79.5006	414.4249	2.3821	0.000107955
ENSMUSG00000028617	*Lrrc42*	146.8690	763.5823	2.3783	0.002460928
ENSMUSG00000055116	*Arntl*	50.2265	260.6913	2.3758	0.002694955
ENSMUSG00000031616	*Ednra*	54.8560	283.9479	2.3719	0.007493832
ENSMUSG00000046688	*Tifa*	206.1928	1067.1236	2.3717	0.001345177
ENSMUSG00000026069	*Il1rl1*	40019.6783	206549.1424	2.3677	1.56E-08
ENSMUSG00000030427	*Lilra6*	65.8811	339.3608	2.3649	0.000842186
ENSMUSG00000029925	*Tbxas1*	2192.4879	11223.1476	2.3558	1.22E-07
ENSMUSG00000032741	*Tpcn1*	257.6274	1306.3639	2.3422	1.83E-06
ENSMUSG00000035847	*Ids*	984.5359	4983.7904	2.3397	2.55E-07
ENSMUSG00000040488	*Ltbp4*	50.9437	256.8502	2.3340	0.000203219
ENSMUSG00000028273	*Pdlim5*	798.2159	4019.2721	2.3321	1.14E-06
ENSMUSG00000024892	*Pcx*	135.5300	681.4763	2.3301	6.82E-06
ENSMUSG00000021203	*Otub2*	68.0289	341.4558	2.3275	0.027700715
ENSMUSG00000055632	*Hmcn2*	7.6706	38.3204	2.3207	0.029907424
ENSMUSG00000028189	*Ctbs*	61.3104	305.9955	2.3193	0.013754758
ENSMUSG00000023008	*Fmnl3*	142.9544	708.3216	2.3088	3.31E-05
ENSMUSG00000027883	*Gpsm2*	544.1708	2695.9671	2.3087	2.09E-06
ENSMUSG00000031808	*Slc27a1*	456.5607	2254.3283	2.3038	1.02E-06
ENSMUSG00000036606	*Plxnb2*	86.6919	426.9404	2.3001	1.55E-05
ENSMUSG00000047250	*Ptgs1*	798.4391	3926.5824	2.2980	4.25E-07
ENSMUSG00000020658	*Efr3b*	18.2591	89.3880	2.2915	0.038678738
ENSMUSG00000029372	*Ppbp*	1415.9789	6920.6642	2.2891	8.32E-06
ENSMUSG00000002365	*Snx9*	150.3665	734.8748	2.2890	8.21E-05
ENSMUSG00000037706	*Cd81*	3194.2411	15602.3979	2.2882	1.83E-07

ENSMUSG00000018378	*Cuedc1*	336.7107	1642.3035	2.2861	1.81E-05
ENSMUSG00000043051	*Disc1*	21.9365	105.8244	2.2703	0.029327291
ENSMUSG00000061666	*Gdpd1*	80.9011	385.0604	2.2509	0.010410409
ENSMUSG00000030530	*Furin*	4742.5453	22524.5515	2.2478	1.08E-07
ENSMUSG00000054892	*Txk*	95.1539	451.8276	2.2474	0.001061578
ENSMUSG00000033306	*Lpp*	758.7510	3582.9037	2.2394	8.46E-07
ENSMUSG00000025429	*Pstpip2*	134.4490	632.0650	2.2330	0.002812603
ENSMUSG00000063415	*Cyp26b1*	38.6612	181.5529	2.2314	0.022572611
ENSMUSG00000010021	*Kif19a*	29.5665	138.6396	2.2293	0.023759088
ENSMUSG00000039197	*Adk*	2707.8084	12573.3218	2.2152	3.17E-07
ENSMUSG00000040612	*Ildr2*	1270.8975	5900.3362	2.2149	4.77E-07
ENSMUSG00000020644	*Id2*	4170.6341	19355.5862	2.2144	6.97E-07
ENSMUSG00000062593	*Lilrb4*	6212.3179	28771.0452	2.2114	1.86E-07
ENSMUSG00000033769	*Exoc6b*	172.0036	792.5687	2.2041	0.000720242
ENSMUSG00000054793	*Cadm4*	95.2313	438.3527	2.2026	0.004117568
ENSMUSG00000023809	*Rps6ka2*	61.6313	281.8951	2.1934	0.005853616
ENSMUSG00000025473	*Adam8*	316.8719	1447.8803	2.1920	5.34E-06
ENSMUSG00000006587	*Snai3*	58.6249	265.2104	2.1776	0.040476745
ENSMUSG00000029919	*Hpgds*	5870.3816	26503.4668	2.1747	4.26E-07
ENSMUSG00000024621	*Csf1r*	38.1169	171.7514	2.1718	0.010559025
ENSMUSG00000037944	*Ccr7*	278.4148	1245.6022	2.1615	0.000128607
ENSMUSG00000038712	*Fam63a*	898.9739	3966.6818	2.1416	2.76E-06
ENSMUSG00000034993	*Vat1*	1668.2318	7265.9515	2.1228	1.47E-06
ENSMUSG00000022353	*Mtss1*	463.1828	2010.1973	2.1177	5.87E-06
ENSMUSG00000022479	*Vdr*	169.1838	731.1590	2.1116	0.03526083
ENSMUSG00000021703	*Serinc5*	98.7579	425.9235	2.1086	0.003583882
ENSMUSG00000040009	*Gnaz*	1778.7179	7663.7177	2.1072	1.94E-06
ENSMUSG00000000934	*Top1mt*	42.8380	183.4475	2.0984	0.020365063
ENSMUSG00000003518	*Dusp3*	1159.4832	4951.9995	2.0945	4.45E-05
ENSMUSG00000020785	*Camkk1*	98.9336	422.2845	2.0937	0.005071019
ENSMUSG00000038807	*Rap1gap2*	107.6337	459.2642	2.0932	0.004963014
ENSMUSG00000036185	*Ng23*	1703.7740	7241.0653	2.0875	1.66E-05
ENSMUSG00000026923	*Notch1*	14.5038	60.9279	2.0707	0.01673038
ENSMUSG00000041439	*Mfsd6*	728.2431	3041.6347	2.0624	5.12E-06
ENSMUSG00000026107	*Obfc2a*	621.4943	2592.2912	2.0604	0.000178838
ENSMUSG00000022305	*Lrp12*	1708.0589	7093.1103	2.0541	1.43E-06
ENSMUSG00000026437	*Cdk18*	162.3940	670.6004	2.0460	0.001127854
ENSMUSG00000030108	*Slc6a13*	181.9134	748.7760	2.0413	0.001218842
ENSMUSG00000071714	*Csf2rb2*	4773.7578	19648.5506	2.0412	1.02E-06
ENSMUSG00000034652	*Cd300a*	1110.8311	4558.4393	2.0369	1.21E-05
ENSMUSG00000063077	*Kif1b*	751.8835	3083.7079	2.0361	3.05E-06
ENSMUSG00000029759	*Pon3*	179.2775	733.8191	2.0332	0.01202227
ENSMUSG00000020846	*Fam101b*	287.1426	1174.1004	2.0317	0.002133276
ENSMUSG00000037161	*4930583H14Rik*	75.5561	306.0834	2.0183	0.043762471
ENSMUSG00000009569	*Mkl2*	551.7459	2217.8754	2.0071	1.91E-05
ENSMUSG00000037979	*Ccdc92*	93.3424	375.1116	2.0067	0.045500993
ENSMUSG00000005947	*Itgae*	623.3101	2499.1470	2.0034	5.39E-06

312 genes upregulated >2-fold in EML cells transduced with ASXL1(1-479)+BAP1 (P<0.05, DE-seq)

ENSEML ID	*Gene name*	*MiG empty expression*	*ASXL1(1-479)+BAP1*	*log2 fold-change*	*p-value*
ENSMUSG00000028927	*Padi2*	491.186185	1951.70218	1.9904	2.22E-05
ENSMUSG00000021256	*Vash1*	532.6233381	2112.061918	1.9875	0.000111165
ENSMUSG00000027544	*Nfatc2*	296.5982235	1175.284362	1.9864	6.59E-05
ENSMUSG00000032578	*Cish*	1403.155078	5550.701812	1.9840	0.000101319
ENSMUSG00000022010	*Tsc22d1*	738.384268	2918.782546	1.9829	3.30E-05
ENSMUSG00000001763	*Tspan33*	197.3611825	778.5002091	1.9799	0.004086184
ENSMUSG00000028926	*Cdk14*	316.6530139	1242.338773	1.9721	0.00068769
ENSMUSG00000056529	*Ptafr*	352.1603775	1377.792553	1.9681	0.000414716
ENSMUSG00000039953	*Clstn1*	1822.964547	7116.237238	1.9648	3.40E-06
ENSMUSG00000026343	*Gpr39*	137.461869	533.9953301	1.9578	0.003290923
ENSMUSG00000071379	*Hpcal1*	128.2027794	495.5928538	1.9507	0.038256523
ENSMUSG00000038260	*Trpm4*	54.42419873	210.3790338	1.9507	0.004166563
ENSMUSG00000062373	*Tmem65*	422.9595969	1632.154255	1.9482	0.000874181
ENSMUSG00000051554	*Gm9853*	1470.841998	5669.361209	1.9465	0.017069044
ENSMUSG00000066621	*Tecpr1*	204.8488367	787.6814029	1.9431	7.49E-05
ENSMUSG00000023926	*Rhag*	80.59408209	308.3431228	1.9358	0.017906415
ENSMUSG00000001761	*Smo*	945.2106925	3612.144663	1.9341	1.01E-05
ENSMUSG00000027463	*Slc52a3*	77.13353324	294.1015716	1.9309	0.033039782
ENSMUSG00000021822	*Plau*	6704.075668	25523.98711	1.9287	3.99E-06
ENSMUSG00000035095	*Fam167a*	446.5272991	1696.500536	1.9257	0.001129265
ENSMUSG00000020383	*Il13*	249.3201532	940.9657028	1.9161	0.023483738
ENSMUSG00000020647	*Ncoa1*	150.6103956	568.2508603	1.9157	0.000125562
ENSMUSG00000022272	*Myo10*	157.2510302	592.4572228	1.9136	0.001625722
ENSMUSG00000026482	*Rgl1*	421.590236	1587.582402	1.9129	8.51E-05
ENSMUSG00000020782	*Llgl2*	107.4216366	402.0996671	1.9043	0.032964129
ENSMUSG00000022157	*Mcpt8*	3830.678286	14269.41878	1.8973	1.15E-05
ENSMUSG00000021665	*Hexb*	2341.365446	8691.917423	1.8923	9.44E-06
ENSMUSG00000047181	*Samd14*	522.4885603	1936.155247	1.8897	0.000461314
ENSMUSG00000032733	*Snx33*	201.1637107	742.8238131	1.8846	0.000888202
ENSMUSG00000026748	*Plxdc2*	1277.563634	4694.295503	1.8775	2.15E-05
ENSMUSG00000038368	*BC057079*	144.4348943	526.4102403	1.8658	0.000310452
ENSMUSG00000026700	*Tnfsf4*	163.2880383	593.6414827	1.8622	0.029907638
ENSMUSG00000060671	*Atp8b2*	1463.031812	5315.822238	1.8613	2.06E-05
ENSMUSG00000025357	*Dgka*	85.86659213	311.880226	1.8608	0.005848545
ENSMUSG00000021196	*Pfkp*	2633.536326	9466.969975	1.8459	1.53E-05
ENSMUSG00000027663	*Zmat3*	1639.303889	5877.631357	1.8422	4.87E-05
ENSMUSG00000051578	*Cmtm4*	269.636743	958.5667847	1.8299	0.009293056
ENSMUSG00000034394	*Lif*	853.5582424	3024.61299	1.8252	0.000636195
ENSMUSG00000027435	*Cd93*	5465.005782	19344.72091	1.8236	1.16E-05
ENSMUSG00000060477	*Irak2*	192.1019892	672.4979637	1.8077	0.004275474
ENSMUSG00000039813	*Tbc1d2*	235.2440232	823.4664693	1.8076	0.000991921

ENSMUSG00000058818	*Lilrb3*	483.9451792	1693.695571	1.8073	8.98E-05
ENSMUSG00000040314	*Ctsg*	17047.79979	59505.38814	1.8034	1.35E-05
ENSMUSG00000038668	*Lpar1*	148.0337217	513.2743331	1.7938	0.029989921
ENSMUSG00000025477	*Inpp5a*	199.6814518	689.7020031	1.7883	0.004190266
ENSMUSG00000025875	*Tspan17*	143.1397006	493.972969	1.7870	0.045197707
ENSMUSG00000027068	*Dhrs9*	771.5101261	2661.62071	1.7865	0.000283685
ENSMUSG00000070469	*Adamtsl3*	172.3900826	593.2361898	1.7829	0.001114308
ENSMUSG00000000562	*Adora3*	3907.940353	13359.48483	1.7734	4.02E-05
ENSMUSG00000013419	*Zfp651*	59.7365159	201.8914434	1.7569	0.024040137
ENSMUSG00000007891	*Ctsd*	87739.60804	296062.9557	1.7546	1.84E-05
ENSMUSG00000032540	*Abhd5*	451.9583736	1524.530853	1.7541	0.003102917
ENSMUSG00000021978	*Extl3*	546.3155215	1839.256805	1.7513	0.00010678
ENSMUSG00000031162	*Gata1*	958.6372729	3224.687287	1.7501	0.000565723
ENSMUSG00000066152	*Slc31a2*	1660.410001	5582.058989	1.7493	0.000428935
ENSMUSG00000027955	*Fam198b*	6814.681604	22761.20594	1.7399	3.55E-05
ENSMUSG00000022667	*Cd200r1*	1022.422867	3407.308411	1.7366	0.000362515
ENSMUSG00000032754	*Slc24a6*	366.9005125	1219.913921	1.7333	0.001548412
ENSMUSG00000038872	*Zfhx3*	106.5135642	353.8912415	1.7323	0.000183366
ENSMUSG00000022836	*Mylk*	157.1936328	521.8785806	1.7312	0.001244948
ENSMUSG00000035759	*Bbs10*	521.9309561	1732.621908	1.7310	0.000206363
ENSMUSG00000034903	*Cobll1*	118.0831565	390.6494797	1.7261	0.003409462
ENSMUSG00000020029	*Nudt4*	2771.968504	9147.995198	1.7225	0.000141764
ENSMUSG00000034403	*Pja1*	538.5328464	1776.596949	1.7220	0.000398376
ENSMUSG00000031790	*Mmp15*	248.5611877	817.6347147	1.7179	0.001253172
ENSMUSG00000022265	*Ank*	1136.574965	3714.999188	1.7087	0.00013676
ENSMUSG00000052544	*St6galnac3*	540.8335829	1757.952873	1.7006	0.001391086
ENSMUSG00000057329	*Bcl2*	3162.088959	10277.91729	1.7006	0.000104763
ENSMUSG00000023009	*Nckap5l*	177.2264727	575.7299003	1.6998	0.005567583
ENSMUSG00000005533	*Igf1r*	916.0086904	2965.162782	1.6947	7.44E-05
ENSMUSG00000017740	*Slc12a5*	127.0616386	410.4709679	1.6918	0.009795013
ENSMUSG00000025352	*Gdf11*	615.829173	1989.162011	1.6916	0.000610418
ENSMUSG00000026879	*Gsn*	7303.774222	23548.27212	1.6889	4.35E-05
ENSMUSG00000041957	*Pkp2*	190.3755515	610.9050322	1.6821	0.005559154
ENSMUSG00000063193	*Cd300lb*	217.5689581	696.8146245	1.6793	0.024566708
ENSMUSG00000050390	*C77080*	691.3252818	2208.498173	1.6756	0.000263757
ENSMUSG00000025856	*Pdgfa*	204.7992601	654.1975932	1.6755	0.038192095
ENSMUSG00000015944	*Gatsl2*	111.3065103	355.2201401	1.6742	0.047385695
ENSMUSG00000021108	*Prkch*	202.3225178	643.6139178	1.6695	0.003287602
ENSMUSG00000020395	*Itk*	354.4109725	1115.573413	1.6543	0.001647414
ENSMUSG00000050271	*D8Ertd82e*	92.85546555	292.0375312	1.6531	0.004146722
ENSMUSG00000046324	*Ermp1*	1391.273313	4368.567291	1.6508	0.000107793
ENSMUSG00000044092	*C130050O18Rik*	196.4623566	616.8244813	1.6506	0.013919536
ENSMUSG00000016487	*Ppfibp1*	671.5726184	2097.619814	1.6431	0.0004803
ENSMUSG00000043017	*Ptgir*	606.1476481	1891.846149	1.6421	0.00157069
ENSMUSG00000022817	*Itgb5*	159.336606	496.7446917	1.6404	0.003729147
ENSMUSG00000035769	*Xylb*	91.11396609	282.9222401	1.6347	0.047415709
ENSMUSG00000054252	*Fgfr3*	89.14420931	276.761246	1.6344	0.024090549
ENSMUSG00000074604	*Mgst2*	595.0135186	1846.35137	1.6337	0.016827159

ENSMUSG00000054008	*Ndst1*	741.8891312	2300.229553	1.6325	0.000360858
ENSMUSG00000064105	*Cnnm2*	335.2542249	1035.718697	1.6273	0.000773238
ENSMUSG00000071454	*Dtnb*	671.9237045	2068.536258	1.6222	0.000686886
ENSMUSG00000001025	*S100a6*	24798.73897	75918.60578	1.6142	0.000112673
ENSMUSG00000002058	*Unc119*	747.0453595	2286.416554	1.6138	0.002476204
ENSMUSG00000047821	*Trim16*	543.4174755	1659.563333	1.6107	0.000989423
ENSMUSG00000032038	*St3gal4*	3571.531803	10892.69828	1.6087	0.000165548
ENSMUSG00000044231	*Nhlrc1*	103.6620179	315.7966195	1.6071	0.048037544
ENSMUSG00000050721	*Plekho2*	1210.997411	3675.474257	1.6017	0.000329717
ENSMUSG00000021451	*Sema4d*	2780.963092	8395.40348	1.5940	0.000133025
ENSMUSG00000031642	*Sh3rf1*	125.7457097	379.4258535	1.5933	0.011043685
ENSMUSG00000040722	*Scamp5*	307.4466312	927.2576453	1.5926	0.017370134
ENSMUSG00000020642	*Rnf144a*	1139.991993	3427.914318	1.5883	0.000835728
ENSMUSG00000071713	*Csf2rb*	11000.62379	33077.74104	1.5883	0.000107009
ENSMUSG00000024063	*Lbh*	5121.43649	15370.62456	1.5856	0.000432145
ENSMUSG00000030137	*Tuba8*	1899.361771	5696.989796	1.5847	0.000268036
ENSMUSG00000034825	*Nrip3*	411.209762	1231.93798	1.5830	0.009027389
ENSMUSG00000044716	*Dok7*	1245.074887	3715.77717	1.5774	0.000481944
ENSMUSG00000037348	*Paqr7*	1472.103431	4388.029272	1.5757	0.000839401
ENSMUSG00000070348	*Ccnd1*	7612.498512	22653.08913	1.5733	0.000164765
ENSMUSG00000036902	*Neto2*	324.3998095	960.3932671	1.5659	0.003202703
ENSMUSG00000040111	*Gramd1b*	1358.764293	4009.820044	1.5612	0.000288853
ENSMUSG00000026826	*Nr4a2*	124.0730353	364.8112383	1.5560	0.021144558
ENSMUSG00000028538	*St3gal3*	394.385714	1159.579733	1.5559	0.007221789
ENSMUSG00000039046	*Usp6nl*	342.02433	1001.256751	1.5496	0.001471
ENSMUSG00000030103	*Bhlhe40*	2435.125617	7124.14888	1.5487	0.000470084
ENSMUSG00000005125	*Ndrg1*	36702.96337	107056.993	1.5444	0.000167258
ENSMUSG00000000278	*Scpep1*	295.0589976	857.0056547	1.5383	0.007436446
ENSMUSG00000043252	*Tmem64*	2509.382654	7230.899616	1.5268	0.000407696
ENSMUSG00000066684	*Pilrb1*	955.9514426	2738.822645	1.5185	0.003008924
ENSMUSG00000041773	*Enc1*	597.6440647	1709.12814	1.5159	0.00136047
ENSMUSG00000041977	*Arhgef11*	140.2848506	398.5418948	1.5064	0.005282901
ENSMUSG00000015437	*Gzmb*	171322.6786	484335.2984	1.4993	0.000225108
ENSMUSG00000058672	*Tubb2a*	787.332161	2196.349257	1.4801	0.001804398
ENSMUSG00000021322	*Aoah*	397.3584819	1108.280914	1.4798	0.008236747
ENSMUSG00000066682	*Pilrb2*	1011.751988	2815.275822	1.4764	0.003652279
ENSMUSG00000089672	*Gp49a*	6128.32374	17034.02153	1.4749	0.000451034
ENSMUSG00000025017	*Pik3ap1*	570.6525156	1579.639639	1.4689	0.001576918
ENSMUSG00000020696	*Rffl*	644.2928749	1777.787599	1.4643	0.004671526
ENSMUSG00000038145	*Snrk*	888.9069381	2446.949031	1.4609	0.002491442
ENSMUSG00000036698	*Eif2c2*	7531.887448	20721.43037	1.4600	0.000362228
ENSMUSG00000003500	*Impdh1*	457.0418749	1252.903105	1.4549	0.006490043
ENSMUSG00000026932	*Nacc2*	363.669004	996.4523023	1.4542	0.0085298
ENSMUSG00000037940	*Inpp4b*	2430.069285	6656.568832	1.4538	0.000524538
ENSMUSG00000027583	*Zbtb46*	298.7561363	816.4380639	1.4504	0.016572108
ENSMUSG00000020173	*Cobl*	2712.558601	7376.094978	1.4432	0.000585815
ENSMUSG00000059149	*Mfsd4*	171.0270694	463.7217247	1.4390	0.03567132
ENSMUSG00000048232	*Fbxo10*	104.8221725	284.1571895	1.4387	0.025051447

ENSMUSG00000042349	*Ikbke*	244.8606487	663.3087005	1.4377	0.013292758
ENSMUSG00000003746	*Man1a*	2159.0808	5845.948197	1.4370	0.000752697
ENSMUSG00000013089	*Etv5*	1946.466688	5264.286899	1.4354	0.001015718
ENSMUSG00000027297	*Ltk*	260.067536	702.9889313	1.4346	0.006066356
ENSMUSG00000045216	*Hs6st1*	1434.41962	3875.996028	1.4341	0.001369205
ENSMUSG00000015053	*Gata2*	2878.451536	7776.427014	1.4338	0.000718748
ENSMUSG00000021257	*Angel1*	91.00923739	244.8949948	1.4281	0.04956612
ENSMUSG00000071547	*Nt5dc2*	835.7681634	2247.882289	1.4274	0.002954123
ENSMUSG00000022475	*Hdac7*	215.528015	576.6954549	1.4199	0.0089972
ENSMUSG00000003228	*Grk5*	121.8983896	325.9478352	1.4190	0.038877867
ENSMUSG00000030748	*Il4ra*	6792.530487	18138.15931	1.4170	0.000617692
ENSMUSG00000020231	*Dip2a*	310.9229854	827.4841996	1.4122	0.002013379
ENSMUSG00000027762	*Sucnr1*	998.0512229	2652.91469	1.4104	0.003479328
ENSMUSG00000036046	*5031439G07Rik*	3262.287371	8665.961878	1.4095	0.00087296
ENSMUSG00000000290	*Itgb2*	5909.344736	15672.89506	1.4072	0.000781134
ENSMUSG00000020841	*Cpd*	1295.435862	3430.061977	1.4048	0.000814991
ENSMUSG00000034761	*Map4k5*	311.3249064	822.0949441	1.4009	0.005025177
ENSMUSG00000025702	*8-Mar*	8531.409326	22490.51953	1.3985	0.000778037
ENSMUSG00000075702	*Selm*	693.9454351	1825.654316	1.3955	0.024486112
ENSMUSG00000024095	*Hnrpll*	989.1653404	2595.427443	1.3917	0.001933172
ENSMUSG00000021624	*Cd180*	4158.150091	10907.28567	1.3913	0.001464648
ENSMUSG00000021939	*Ctsb*	11503.97701	29972.65545	1.3815	0.000786752
ENSMUSG00000039031	*Arhgap18*	4419.586985	11508.53269	1.3807	0.00112996
ENSMUSG00000048537	*Phldb1*	257.6558007	670.5720994	1.3799	0.007636415
ENSMUSG00000034361	*Cpne2*	1703.472673	4429.068298	1.3785	0.001447767
ENSMUSG00000034271	*Jdp2*	833.8791169	2167.463977	1.3781	0.016105849
ENSMUSG00000057469	*E2f6*	1720.088896	4458.810142	1.3742	0.005490299
ENSMUSG00000037580	*Gch1*	3059.524752	7923.736425	1.3729	0.001810254
ENSMUSG00000037754	*Ppp1r16b*	839.4825031	2173.501885	1.3724	0.002994012
ENSMUSG00000027298	*Tyro3*	608.261704	1573.166076	1.3709	0.002479988
ENSMUSG00000020272	*Stk10*	708.3128354	1831.446678	1.3705	0.001963995
ENSMUSG00000052302	*Tbc1d30*	109.8259319	282.713279	1.3641	0.038091064
ENSMUSG00000061175	*Fnip2*	2408.655096	6189.147206	1.3615	0.001017859
ENSMUSG00000024014	*Pim1*	1869.758419	4790.61517	1.3574	0.002032611
ENSMUSG00000068115	*Ninl*	262.3732625	671.8253056	1.3565	0.009749878
ENSMUSG00000079186	*Gzmc*	899.2666784	2301.876537	1.3560	0.008327167
ENSMUSG00000025969	*Nrp2*	663.4244997	1698.147526	1.3560	0.002446698
ENSMUSG00000047557	*Lxn*	1035.793759	2642.768859	1.3513	0.008195617
ENSMUSG00000004105	*Angptl2*	4755.901839	12095.8821	1.3467	0.001201585
ENSMUSG00000030844	*Rgs10*	4062.953064	10316.25767	1.3443	0.002978793
ENSMUSG00000053338	*Tarm1*	421.5497315	1070.021763	1.3439	0.023200515
ENSMUSG00000004071	*5730403B10Rik*	439.8779807	1114.782895	1.3416	0.041492897
ENSMUSG00000021306	*Gpr137b*	726.8163808	1839.811535	1.3399	0.006584957
ENSMUSG00000021365	*Nedd9*	733.0859625	1854.589272	1.3390	0.002764174
ENSMUSG00000028680	*Plk3*	135.9166984	343.4010354	1.3372	0.041783382
ENSMUSG00000032802	*Srxn1*	2725.429551	6881.702918	1.3363	0.004406067
ENSMUSG00000024535	*Snx24*	603.2054558	1522.546826	1.3358	0.031888043
ENSMUSG00000025025	*Mxi1*	1233.350184	3110.278368	1.3345	0.006478606

ENSMUSG00000038764	*Ptpn3*	1630.284344	4109.298216	1.3338	0.003401536
ENSMUSG00000013698	*Pea15a*	1074.530561	2707.251254	1.3331	0.025471523
ENSMUSG00000034875	*Nudt19*	219.9389904	553.7756934	1.3322	0.048583363
ENSMUSG00000062082	*Cd200r4*	5637.769453	14157.81072	1.3284	0.001643819
ENSMUSG00000068747	*Sort1*	189.9919403	472.6138278	1.3147	0.024831617
ENSMUSG00000031530	*Dusp4*	6279.037131	15585.59551	1.3116	0.001573766
ENSMUSG00000031827	*Cotl1*	6468.064956	16042.76637	1.3105	0.00249984
ENSMUSG00000079037	*Prnp*	10735.0143	26578.85993	1.3080	0.001582739
ENSMUSG00000029135	*Fosl2*	1400.59308	3436.122586	1.2947	0.005056265
ENSMUSG00000030605	*Mfge8*	8612.504086	21112.30739	1.2936	0.001648445
ENSMUSG00000033220	*Rac2*	15055.5112	36899.96361	1.2933	0.001756089
ENSMUSG00000024511	*Rab27b*	18452.17507	45105.64171	1.2895	0.001688314
ENSMUSG00000027660	*Skil*	281.4994119	688.0290585	1.2893	0.020334416
ENSMUSG00000021552	*Gkap1*	489.4543925	1195.341699	1.2882	0.017194948
ENSMUSG00000027765	*P2ry1*	785.4121586	1898.866555	1.2736	0.009648916
ENSMUSG00000038633	*Degs1*	7638.098633	18433.09877	1.2710	0.00225683
ENSMUSG00000026509	*Capn2*	2873.154225	6925.903038	1.2694	0.002472142
ENSMUSG00000020097	*Sgpl1*	15137.75504	36471.99551	1.2686	0.001959284
ENSMUSG00000062661	*Ncs1*	3549.12076	8536.834887	1.2662	0.00435384
ENSMUSG00000050989	*Sepn1*	1543.151821	3701.741793	1.2623	0.00557321
ENSMUSG00000038172	*Ttc39b*	1077.93801	2569.000597	1.2529	0.008474582
ENSMUSG00000028521	*Slc35d1*	1326.269437	3157.52065	1.2514	0.00896857
ENSMUSG00000032849	*Abcc4*	373.5973374	889.1354608	1.2509	0.006506177
ENSMUSG00000024772	*Ehd1*	443.4767307	1047.896933	1.2406	0.015587004
ENSMUSG00000048120	*Entpd1*	5533.570191	13068.31407	1.2398	0.002831717
ENSMUSG00000035469	*Rcbtb1*	833.8994811	1968.803176	1.2394	0.017802935
ENSMUSG00000035776	*Cd99l2*	2271.248617	5361.976339	1.2393	0.006967848
ENSMUSG00000036989	*Trim3*	231.4278343	546.0733795	1.2385	0.037086092
ENSMUSG00000003178	*Mical3*	257.3950433	605.7766512	1.2348	0.02842919
ENSMUSG00000006362	*Cbfa2t3*	2232.636495	5253.70583	1.2346	0.004014698
ENSMUSG00000029338	*Antxr2*	1358.247461	3168.765697	1.2222	0.006051928
ENSMUSG00000023805	*Synj2*	116.1309644	270.1866763	1.2182	0.043375219
ENSMUSG00000049643	*2310022A10Rik*	276.5650737	641.7512466	1.2144	0.048594678
ENSMUSG00000018774	*Cd68*	399.2840975	926.3101521	1.2141	0.038618648
ENSMUSG00000050108	*Bpifc*	483.940461	1121.883376	1.2130	0.034992212
ENSMUSG00000027074	*Slc43a3*	1219.612788	2826.102932	1.2124	0.017036009
ENSMUSG00000022895	*Ets2*	1070.187366	2476.802973	1.2106	0.010147378
ENSMUSG00000041075	*Fzd7*	658.0866818	1519.69341	1.2074	0.011055384
ENSMUSG00000026014	*Raph1*	443.2903831	1020.437693	1.2029	0.017745214
ENSMUSG00000049076	*Acap2*	1157.767654	2664.292103	1.2024	0.0057059
ENSMUSG00000057069	*Ero1lb*	590.5856401	1353.665612	1.1967	0.016579884
ENSMUSG00000022105	*Rb1*	1022.680485	2340.003378	1.1942	0.005840212
ENSMUSG00000021823	*Vcl*	4603.90334	10486.79002	1.1876	0.004027013
ENSMUSG00000051950	*B3galtl*	518.8131399	1177.744963	1.1827	0.019788319
ENSMUSG00000035891	*Cerk*	4246.536702	9599.288811	1.1766	0.005173784
ENSMUSG00000000184	*Ccnd2*	12270.54087	27688.17423	1.1741	0.004276084
ENSMUSG00000058173	*BC017612*	4154.61025	9356.015795	1.1712	0.021975613
ENSMUSG00000022634	*Yaf2*	1835.691908	4131.838226	1.1705	0.015986065

ENSMUSG00000024533	*Spire1*	704.3452176	1582.019005	1.1674	0.013534762
ENSMUSG00000027506	*Tpd52*	2218.010237	4977.838242	1.1663	0.013107587
ENSMUSG00000038459	*Fam108c*	1350.904889	3027.419199	1.1642	0.012844856
ENSMUSG00000033066	*Gas7*	4088.309029	9143.990684	1.1613	0.00568605
ENSMUSG00000018001	*Cyth3*	3367.641913	7522.173051	1.1594	0.009739787
ENSMUSG00000042680	*Fam59a*	195.0828716	434.8330074	1.1564	0.036528565
ENSMUSG00000035615	*Frmpd1*	847.9496396	1886.563166	1.1537	0.007816665
ENSMUSG00000017765	*Slc12a4*	690.48601	1530.809234	1.1486	0.011866045
ENSMUSG00000022377	*Asap1*	326.8458143	724.2848089	1.1479	0.019072876
ENSMUSG00000061689	*Dlgap4*	416.96491	921.426772	1.1439	0.03372517
ENSMUSG00000050708	*Ftl1*	19646.68129	43295.4788	1.1399	0.005505149
ENSMUSG00000031758	*Cdyl2*	385.0025468	847.6756833	1.1386	0.034363418
ENSMUSG00000038831	*Ralgps1*	615.5746204	1355.225112	1.1385	0.023496219
ENSMUSG00000022066	*Entpd4*	1022.552172	2247.804954	1.1363	0.015818261
ENSMUSG00000031709	*Tbc1d9*	132.8631446	291.7858451	1.1350	0.048853119
ENSMUSG00000002608	*Ccdc97*	504.5659191	1107.457047	1.1341	0.040514744
ENSMUSG00000002103	*Acp2*	1345.316959	2944.465587	1.1301	0.015551736
ENSMUSG00000026389	*Steap3*	2179.834022	4770.157048	1.1298	0.00865979
ENSMUSG00000044768	*D1Ertd622e*	827.7226814	1810.517415	1.1292	0.033176832
ENSMUSG00000037815	*Ctnna1*	3301.633817	7209.46766	1.1267	0.006277581
ENSMUSG00000028042	*Zbtb7b*	799.3052975	1744.52381	1.1260	0.025577362
ENSMUSG00000028581	*Laptm5*	32064.95132	69908.21916	1.1245	0.005651447
ENSMUSG00000030096	*Slc6a6*	10192.10031	22159.50922	1.1205	0.005896259
ENSMUSG00000038366	*Lasp1*	19583.33016	42555.01428	1.1197	0.007555949
ENSMUSG00000029050	*Ski*	851.1944943	1848.941133	1.1191	0.013050273
ENSMUSG00000057963	*Itpk1*	381.8749773	827.9177738	1.1164	0.047804812
ENSMUSG00000000682	*Cd52*	10213.78838	22128.25865	1.1154	0.011434392
ENSMUSG00000031987	*Egln1*	1947.284836	4198.133909	1.1083	0.011766479
ENSMUSG00000056498	*Tmem154*	1201.158519	2578.984071	1.1024	0.036246396
ENSMUSG00000050675	*Gp1ba*	625.2080428	1336.162611	1.0957	0.018438385
ENSMUSG00000036026	*Tmem63b*	895.9656142	1912.576278	1.0940	0.028464265
ENSMUSG00000026490	*Cdc42bpa*	1123.833343	2396.753921	1.0927	0.010227109
ENSMUSG00000053581	*Zfand2a*	2785.468629	5940.385092	1.0926	0.021043696
ENSMUSG00000028480	*Glipr2*	4866.498167	10373.81547	1.0920	0.015874695
ENSMUSG00000029364	*Wsb2*	4724.615349	10053.0851	1.0894	0.009126596
ENSMUSG00000022914	*Brwd1*	237.2778619	504.2381916	1.0875	0.023808625
ENSMUSG00000042444	*Fam63b*	3245.838394	6886.61932	1.0852	0.009343909
ENSMUSG00000030123	*Plxnd1*	1858.007251	3934.177832	1.0823	0.010307674
ENSMUSG00000026872	*Zeb2*	2192.141927	4632.840281	1.0796	0.011374537
ENSMUSG00000029467	*Atp2a2*	7822.643804	16485.2009	1.0754	0.008003978
ENSMUSG00000027678	*Ncoa3*	1162.486095	2449.423545	1.0752	0.010565601
ENSMUSG00000020300	*Cpeb4*	1243.494072	2619.654282	1.0750	0.015094426
ENSMUSG00000052160	*Pld4*	417.1407491	878.4759056	1.0745	0.043259629
ENSMUSG00000028737	*Aldh4a1*	1137.655952	2392.913496	1.0727	0.016705886
ENSMUSG00000035392	*Dennd1a*	823.2729769	1728.710912	1.0703	0.021490265
ENSMUSG00000030352	*Tspan9*	3274.321083	6868.176496	1.0687	0.018690006
ENSMUSG00000026123	*Plekhb2*	4403.973439	9226.211952	1.0669	0.016165273
ENSMUSG00000034640	*Tiparp*	1752.200461	3668.929665	1.0662	0.015726519

ENSMUSG00000035828	*Pim3*	1006.448928	2105.60753	1.0650	0.029379948
ENSMUSG00000029103	*Lrpap1*	28821.17186	60201.88016	1.0627	0.009139199
ENSMUSG00000056069	*Fam105a*	8779.790154	18338.8296	1.0626	0.009979652
ENSMUSG00000067825	*Pex26*	1691.543334	3524.175813	1.0589	0.025370786
ENSMUSG00000022221	*Ripk3*	992.6321394	2066.014433	1.0575	0.023461536
ENSMUSG00000029470	*P2rx4*	5488.11178	11402.26019	1.0549	0.011460348
ENSMUSG00000022309	*Angpt1*	10148.99197	21013.08865	1.0500	0.010023809
ENSMUSG00000024597	*Slc12a2*	574.1584427	1185.13399	1.0455	0.018836016
ENSMUSG00000049871	*Nlrc3*	915.5310448	1884.996902	1.0419	0.020055385
ENSMUSG00000042978	*Sbk1*	957.2884113	1969.879904	1.0411	0.028356467
ENSMUSG00000038775	*Vill*	1344.477906	2765.825911	1.0407	0.020944454
ENSMUSG00000008318	*Relt*	3581.041526	7360.509596	1.0394	0.020994682
ENSMUSG00000026603	*Smyd2*	1310.490982	2691.238623	1.0382	0.022304584
ENSMUSG00000003153	*Slc2a3*	9970.867167	20460.32264	1.0370	0.012847295
ENSMUSG00000002489	*Tiam1*	2247.519047	4602.276393	1.0340	0.012349677
ENSMUSG00000055013	*Agap1*	2200.316799	4505.047085	1.0338	0.01350358
ENSMUSG00000041268	*Dmxl2*	466.5349457	952.2634799	1.0294	0.01640425
ENSMUSG00000024968	*Rcor2*	753.3700332	1534.82017	1.0266	0.038827852
ENSMUSG00000071669	*Snx29*	1219.689717	2478.008324	1.0227	0.029439321
ENSMUSG00000004317	*Clcn5*	2625.534528	5328.973414	1.0212	0.015365054
ENSMUSG00000023800	*Tiam2*	364.7000201	740.1310584	1.0211	0.02716
ENSMUSG00000020091	*Eif4ebp2*	2372.591859	4810.537184	1.0197	0.042327738
ENSMUSG00000002699	*Lcp2*	5118.454467	10372.44263	1.0190	0.013605405
ENSMUSG00000039308	*Ndst2*	12800.67341	25912.13952	1.0174	0.011811846
ENSMUSG00000028277	*Ube2j1*	2504.108455	5068.82957	1.0174	0.023998984
ENSMUSG00000042225	*Ammecr1*	749.0073466	1515.571808	1.0168	0.046991267
ENSMUSG00000040451	*Sgms1*	1260.66444	2549.393143	1.0160	0.033857145
ENSMUSG00000032727	*Mier3*	1455.370062	2941.973864	1.0154	0.02120949
ENSMUSG00000024664	*Fads3*	973.5358363	1964.936187	1.0132	0.030458361
ENSMUSG00000040322	*Slc25a24*	2185.035991	4398.880701	1.0095	0.019238627
ENSMUSG00000042599	*Jhdm1d*	967.6145688	1943.729801	1.0063	0.020801616

138 genes downregulated >2-fold in EML cells transduced with ASXL1(1-479)+BAP1 (P<0.05, DE-seq)

ENSEML ID	*Gene name*	*MiG empty expression*	*ASXL1(1-479)+BAP1*	*log2 fold-change*	*p-value*
ENSMUSG00000051998	*Lax1*	2419.5991	614.0073	-1.9784	4.80E-05
ENSMUSG00000016494	*Cd34*	52306.5937	13405.0576	-1.9642	2.32E-06
ENSMUSG00000028525	*Pde4b*	2289.8362	595.2162	-1.9438	4.46E-05
ENSMUSG00000024544	*D18Ertd653e*	6857.4623	1785.3447	-1.9415	1.50E-05
ENSMUSG00000025163	*Cd7*	1418.6065	372.0769	-1.9308	0.002026348
ENSMUSG00000024663	*Rab3il1*	1333.9062	353.0450	-1.9177	0.002961638
ENSMUSG00000056144	*Trim34a*	943.0403	251.8010	-1.9050	0.000959818
ENSMUSG00000069874	*Irgm2*	955.9942	260.3642	-1.8765	0.00126053
ENSMUSG00000040710	*St8sia4*	39429.1183	10789.5954	-1.8696	6.92E-06
ENSMUSG00000033032	*Afap1l1*	6901.4450	1983.0485	-1.7992	2.18E-05
ENSMUSG00000019122	*Ccl9*	10651.9202	3119.5520	-1.7717	0.000312938
ENSMUSG00000015533	*Itga2*	238.6095	70.3321	-1.7624	0.005007587

ENSMUSG00000019737	*AI428936*	1058.6486	314.4948	-1.7511	0.005946809
ENSMUSG00000027165	*B230118H07Rik*	710.4423	211.8958	-1.7454	0.028149157
ENSMUSG00000073598	*1700066B19Rik*	1641.7493	493.2570	-1.7348	0.011637295
ENSMUSG00000044037	*Als2cl*	367.5742	111.2647	-1.7240	0.003671421
ENSMUSG00000030341	*Tnfrsf1a*	1010.0505	306.6663	-1.7197	0.008638908
ENSMUSG00000028950	*Tas1r1*	420.0260	127.9976	-1.7144	0.003499998
ENSMUSG00000000392	*Fap*	238.4097	73.0156	-1.7072	0.028460623
ENSMUSG00000032392	*Parp16*	788.2019	242.2608	-1.7020	0.006743779
ENSMUSG00000028015	*Ctso*	564.6511	173.7942	-1.7000	0.019201307
ENSMUSG00000027605	*Acss2*	13404.6164	4137.5124	-1.6959	4.84E-05
ENSMUSG00000040296	*Ddx58*	1873.1828	578.8262	-1.6943	0.000203254
ENSMUSG00000039741	*Bahcc1*	331.0319	102.5800	-1.6902	0.000523935
ENSMUSG00000035208	*Slfn8*	1422.1405	440.7222	-1.6901	0.000462135
ENSMUSG00000053560	*Ier2*	7411.7449	2310.3136	-1.6817	0.000197813
ENSMUSG00000020323	*Prss57*	5165.5063	1637.6961	-1.6572	0.000525101
ENSMUSG00000025213	*Kazald1*	4467.9938	1420.3442	-1.6534	0.000309563
ENSMUSG00000022324	*Matn2*	688.3986	219.9034	-1.6464	0.001259633
ENSMUSG00000031480	*Thsd1*	3909.4039	1248.9732	-1.6462	0.000148124
ENSMUSG00000023044	*Csad*	918.7918	296.4878	-1.6318	0.00297784
ENSMUSG00000050921	*P2ry10*	5511.7578	1789.2033	-1.6232	0.000259702
ENSMUSG00000025790	*Slco3a1*	20315.2719	6689.8094	-1.6025	0.000110913
ENSMUSG00000022508	*Bcl6*	488.5632	161.0131	-1.6014	0.006262295
ENSMUSG00000002325	*Irf9*	5448.5120	1833.0413	-1.5716	0.00032583
ENSMUSG00000060791	*Gmfg*	16023.9941	5409.3121	-1.5667	0.000414935
ENSMUSG00000026638	*Irf6*	490.6942	169.0048	-1.5378	0.050437847
ENSMUSG00000047507	*Baiap3*	169.5764	58.6882	-1.5308	0.027035035
ENSMUSG00000025746	*Il6*	23217.1142	8035.1643	-1.5308	0.000275642
ENSMUSG00000027858	*Tspan2*	6864.8081	2383.7000	-1.5260	0.000741725
ENSMUSG00000025491	*Ifitm1*	114204.3582	40132.4686	-1.5088	0.000249318
ENSMUSG00000040528	*Gm885*	18031.2561	6337.8732	-1.5084	0.000370146
ENSMUSG00000038543	*BC028528*	4622.4424	1628.4699	-1.5051	0.002714287
ENSMUSG00000015355	*Cd48*	70590.2908	25070.8912	-1.4935	0.000279217
ENSMUSG00000059588	*Calcrl*	5059.0718	1820.7229	-1.4744	0.000708947
ENSMUSG00000069516	*Lyz2*	1484.5882	534.7738	-1.4731	0.025854536
ENSMUSG00000022419	*Deptor*	1548.9572	565.0433	-1.4549	0.004235469
ENSMUSG00000064356	*mt-Atp8*	84771.7088	31030.2646	-1.4499	0.000519413
ENSMUSG00000026664	*Phyh*	1238.6209	456.3655	-1.4405	0.009072382
ENSMUSG00000002985	*Apoe*	1089.2784	407.1300	-1.4198	0.026284891
ENSMUSG00000033705	*Stard9*	1311.9538	490.9448	-1.4181	0.001602784
ENSMUSG00000044786	*Zfp36*	1247.2402	472.9893	-1.3989	0.012034461
ENSMUSG00000006179	*Prss16*	376.5753	142.9223	-1.3977	0.04176351
ENSMUSG00000037447	*Arid5a*	2312.2426	879.1549	-1.3951	0.002667245
ENSMUSG00000037826	*Ppm1k*	818.5633	312.3490	-1.3899	0.01972158
ENSMUSG00000039193	*Nlrc4*	446.8585	170.6992	-1.3884	0.01001134
ENSMUSG00000047735	*Samd9l*	4279.3321	1650.8031	-1.3742	0.001182221
ENSMUSG00000053835	*H2-T24*	3344.9786	1291.4907	-1.3730	0.003602919
ENSMUSG00000029333	*Rasgef1b*	8275.2844	3197.8271	-1.3717	0.001174666
ENSMUSG00000033880	*Lgals3bp*	5785.2106	2236.7273	-1.3710	0.002550984

ENSMUSG00000060131	*Atp8b4*	39020.6471	15142.7921	-1.3656	0.000776871
ENSMUSG00000046718	*Bst2*	11149.7560	4341.1146	-1.3609	0.00196894
ENSMUSG00000022686	*B3gnt5*	2441.8492	961.1162	-1.3452	0.004627177
ENSMUSG00000037890	*Wdr19*	173.6878	68.5643	-1.3410	0.036668884
ENSMUSG00000064370	*mt-Cytb*	47923.8216	19168.0973	-1.3220	0.001186521
ENSMUSG00000031755	*Bbs2*	285.9817	114.4445	-1.3213	0.047182829
ENSMUSG00000024349	*Tmem173*	7062.9104	2839.1084	-1.3148	0.002116357
ENSMUSG00000033446	*Lpar6*	3441.9813	1390.2532	-1.3079	0.004395866
ENSMUSG00000028381	*Ugcg*	50061.5194	20332.4258	-1.2999	0.001413475
ENSMUSG00000059336	*Slc14a1*	30003.9205	12268.4367	-1.2902	0.001623027
ENSMUSG00000025464	*Paox*	4382.9487	1795.6561	-1.2874	0.003628602
ENSMUSG00000030671	*Pde3b*	10284.7995	4246.8495	-1.2760	0.001837089
ENSMUSG00000081058	*Hist2h3c2*	1765.9807	730.5452	-1.2734	0.043969961
ENSMUSG00000064357	*mt-Atp6*	16381.5621	6786.5428	-1.2713	0.002575979
ENSMUSG00000005364	*Il5ra*	1136.8523	477.7869	-1.2506	0.019188726
ENSMUSG00000036306	*Lzts1*	477.5136	200.7268	-1.2503	0.037111529
ENSMUSG00000072620	*Slfn2*	1450.5500	613.8912	-1.2405	0.015842057
ENSMUSG00000064345	*mt-Nd2*	28093.8591	11912.7361	-1.2378	0.002547805
ENSMUSG00000029716	*Tfr2*	675.9420	287.4092	-1.2338	0.017895297
ENSMUSG00000030094	*Xpc*	2747.4637	1169.7245	-1.2319	0.004095857
ENSMUSG00000079523	*Tmsb10*	102706.5099	43806.1488	-1.2293	0.003270279
ENSMUSG00000022906	*Parp9*	3364.1560	1437.8633	-1.2263	0.004312136
ENSMUSG00000030428	*Ttyh1*	1447.9942	619.1794	-1.2256	0.014191649
ENSMUSG00000036356	*Csgalnact1*	1510.0591	646.3468	-1.2242	0.013113745
ENSMUSG00000010358	*Ifi35*	3006.0892	1290.6501	-1.2198	0.010432977
ENSMUSG00000001741	*Il16*	4256.5367	1834.0615	-1.2146	0.005391572
ENSMUSG00000040732	*Erg*	4042.2471	1754.1129	-1.2044	0.006114031
ENSMUSG00000027313	*Chac1*	4355.3838	1912.0218	-1.1877	0.011094058
ENSMUSG00000031309	*Rps6ka3*	41885.7574	18393.1587	-1.1873	0.003550546
ENSMUSG00000056758	*Hmga2*	38611.8210	17061.3738	-1.1783	0.004734063
ENSMUSG00000028332	*Hemgn*	3695.2374	1634.4205	-1.1769	0.007155788
ENSMUSG00000090378	*Gm10925*	10966.9578	4857.9945	-1.1747	0.03616375
ENSMUSG00000063439	*B9d2*	5078.7055	2251.7067	-1.1734	0.013088252
ENSMUSG00000064363	*mt-Nd4*	32490.0005	14407.8307	-1.1731	0.003952561
ENSMUSG00000052713	*Zfp608*	1464.2887	652.6902	-1.1657	0.009232042
ENSMUSG00000050147	*F2rl3*	7742.0191	3452.1358	-1.1652	0.005989707
ENSMUSG00000042289	*Hsd3b7*	7884.6835	3525.8253	-1.1611	0.008249789
ENSMUSG00000073002	*Vamp5*	18623.6574	8363.7111	-1.1549	0.008147157
ENSMUSG00000052560	*Cpne8*	1334.0546	599.3787	-1.1543	0.026762707
ENSMUSG00000036814	*Slc6a20a*	793.8250	356.7765	-1.1538	0.038617595
ENSMUSG00000034401	*Spata6*	1375.0958	618.0640	-1.1537	0.03259051
ENSMUSG00000004612	*Nkg7*	36049.1581	16314.9976	-1.1438	0.005872081
ENSMUSG00000024986	*Hhex*	6825.2612	3094.1213	-1.1414	0.011681439
ENSMUSG00000020812	*1810032O08Rik*	90686.1137	41147.3208	-1.1401	0.005444133
ENSMUSG00000027508	*Pag1*	12636.4947	5753.7559	-1.1350	0.00616583
ENSMUSG00000090124	*Ugt1a7c*	3196.0629	1462.6818	-1.1277	0.013301407
ENSMUSG00000053113	*Socs3*	2494.1082	1146.5956	-1.1212	0.027342265
ENSMUSG00000041143	*Tmco4*	1012.6512	468.0839	-1.1133	0.038262525

ENSMUSG00000027478	*Dnmt3b*	3386.1551	1566.9471	-1.1117	0.008939915
ENSMUSG00000049804	*Armcx4*	4211.5533	1954.9195	-1.1072	0.007336136
ENSMUSG00000038147	*Cd84*	17096.6429	7947.8536	-1.1051	0.007835001
ENSMUSG00000026921	*Egfl7*	2975.4077	1394.6374	-1.0932	0.033089289
ENSMUSG00000019947	*Arid5b*	408.6349	192.3182	-1.0873	0.041622766
ENSMUSG00000052837	*Junb*	3243.4608	1528.2995	-1.0856	0.017657738
ENSMUSG00000039697	*Ncoa7*	4718.3138	2225.0174	-1.0845	0.013306635
ENSMUSG00000045273	*Cenph*	5379.8355	2537.8316	-1.0840	0.016033766
ENSMUSG00000064341	*mt-Nd1*	87380.1010	41228.6369	-1.0837	0.007336047
ENSMUSG00000021556	*Golm1*	14941.5805	7089.8081	-1.0755	0.009182169
ENSMUSG00000011008	*Mcoln2*	4145.9494	1970.1188	-1.0734	0.014549767
ENSMUSG00000050075	*Gpr171*	24288.0902	11541.7841	-1.0734	0.008869268
ENSMUSG00000020423	*Btg2*	5752.5182	2734.8504	-1.0727	0.022192849
ENSMUSG00000028378	*Ptgr1*	4559.5559	2176.3632	-1.0670	0.016149208
ENSMUSG00000026520	*Pycr2*	7361.0831	3531.0718	-1.0598	0.013425708
ENSMUSG00000026004	*1110028C15Rik*	1423.5653	684.1838	-1.0571	0.020047934
ENSMUSG00000021236	*Entpd5*	5995.7392	2886.2455	-1.0547	0.013347416
ENSMUSG00000026532	*Spna1*	248.8999	119.9348	-1.0533	0.035024465
ENSMUSG00000040618	*Pck2*	9766.8138	4714.0760	-1.0509	0.010701163
ENSMUSG00000074578	*1500012F01Rik*	1145241.8111	553070.7349	-1.0501	0.009037643
ENSMUSG00000021756	*Il6st*	2396.0638	1165.0052	-1.0403	0.015614216
ENSMUSG00000043557	*Mdga1*	2476.9701	1208.4745	-1.0354	0.018064738
ENSMUSG00000032640	*Chsy1*	3115.8966	1520.3337	-1.0353	0.015469993
ENSMUSG00000060639	*Hist1h4i*	7735.2329	3786.4771	-1.0306	0.030638764
ENSMUSG00000049502	*Dtx3l*	2648.4384	1297.5570	-1.0293	0.017797303
ENSMUSG00000041736	*Tspo*	24635.7697	12076.7458	-1.0285	0.013590179
ENSMUSG00000026785	*Pkn3*	2274.5710	1124.0457	-1.0169	0.02915724
ENSMUSG00000030691	*Fchsd2*	2325.5916	1157.7285	-1.0063	0.021240447
ENSMUSG00000063889	*Crem*	4037.3119	2010.3860	-1.0059	0.02998332
ENSMUSG00000051212	*Gpr183*	5406.0634	2699.0669	-1.0021	0.0208036

84 genes downregulated >4-fold in EML cells transduced with ASXL1(1-479)+BAP1 (P<0.05, DE-seq)

ENSEML ID	***Gene name***	***MiG empty expression***	***ASXL1(1-479)+BAP1***	***log2 fold-change***	***p-value***
ENSMUSG00000023341	*Mx2*	67.9964	0.0000	∞	0.000249991
ENSMUSG00000033355	*Rtp4*	315.3342	1.3526	-7.8650	3.25E-06
ENSMUSG00000028420	*Tmem38b*	64231.5169	542.5163	-6.8875	1.80E-39
ENSMUSG00000035202	*Lars2*	139861.8130	1382.2732	-6.6608	4.27E-39
ENSMUSG00000029561	*Oasl2*	269.3864	3.3001	-6.3510	1.12E-05
ENSMUSG00000030851	*Ldhc*	77.5853	1.2572	-5.9475	0.043102136
ENSMUSG00000032690	*Oas2*	288.9748	5.4329	-5.7331	5.75E-09
ENSMUSG00000025492	*Ifitm3*	257.6658	4.9167	-5.7117	0.006837093
ENSMUSG00000078606	*Gm4070*	40.8868	0.8144	-5.6497	0.000165718
ENSMUSG00000073867	*AA474408*	22115.9090	506.4198	-5.4486	1.22E-25
ENSMUSG00000025014	*Dntt*	95.1659	2.5907	-5.1990	0.00208409
ENSMUSG00000021886	*Gpr65*	306.4135	8.9945	-5.0903	1.57E-05

ENSMUSG00000026285	*Pdcd1*	180.9915	5.8471	-4.9521	0.00212406
ENSMUSG00000041857	*Oosp1*	267.0688	9.1645	-4.8650	0.008679008
ENSMUSG00000034459	*Ifit1*	249.3909	9.4564	-4.7210	1.52E-05
ENSMUSG00000039997	*Ifi203*	826.3555	36.8752	-4.4860	2.82E-11
ENSMUSG00000035042	*Ccl5*	496.6754	25.9072	-4.2609	0.009276349
ENSMUSG00000030921	*Trim30a*	4079.1288	219.1716	-4.2181	2.65E-15
ENSMUSG00000035692	*Isg15*	342.2032	18.8270	-4.1840	0.004498522
ENSMUSG00000006389	*Mpl*	117.4205	6.5984	-4.1534	0.00130426
ENSMUSG00000024677	*Ms4a6b*	217.6855	12.4227	-4.1312	0.005667025
ENSMUSG00000057596	*Trim30d*	612.2110	36.3699	-4.0732	5.63E-07
ENSMUSG00000046774	*8030474K03Rik*	705.0616	44.2643	-3.9935	6.96E-05
ENSMUSG00000020641	*Rsad2*	2929.1043	184.9283	-3.9854	8.07E-13
ENSMUSG00000064215	*Ifi27l1*	1554.6548	107.7142	-3.8513	5.67E-08
ENSMUSG00000025498	*Irf7*	2868.0103	212.2369	-3.7563	8.95E-13
ENSMUSG00000058427	*Cxcl2*	1164.7789	89.3534	-3.7044	0.000260842
ENSMUSG00000078921	*Tgtp2*	253.5923	19.4772	-3.7027	0.000422235
ENSMUSG00000024681	*Ms4a3*	445.0734	37.1269	-3.5835	0.001737938
ENSMUSG00000027556	*Car1*	347.6656	30.5309	-3.5094	0.00794903
ENSMUSG00000046916	*Myct1*	4309.8683	392.3289	-3.4575	2.68E-10
ENSMUSG00000034115	*Scn11a*	98.8791	9.1593	-3.4324	4.27E-05
ENSMUSG00000004698	*Hdac9*	781.9121	76.4453	-3.3545	6.36E-08
ENSMUSG00000055629	*B4galnt4*	36.3729	3.5829	-3.3437	0.044901758
ENSMUSG00000024228	*Nudt12*	195.7961	19.5007	-3.3278	0.003600124
ENSMUSG00000020638	*Cmpk2*	678.2682	68.5643	-3.3063	1.56E-06
ENSMUSG00000053063	*Clec12a*	914.3881	101.0381	-3.1779	4.32E-05
ENSMUSG00000032661	*Oas3*	565.1860	65.8194	-3.1021	1.57E-06
ENSMUSG00000026896	*Ifih1*	206.4195	24.2370	-3.0903	5.84E-05
ENSMUSG00000027400	*Pdyn*	250.4125	30.4332	-3.0406	0.048449249
ENSMUSG00000022014	*Epsti1*	358.5421	44.4822	-3.0108	0.003727825
ENSMUSG00000000753	*Serpinf1*	8899.6408	1158.0193	-2.9421	5.88E-10
ENSMUSG00000035000	*Dpp4*	81.6089	10.6356	-2.9398	0.018972273
ENSMUSG00000044629	*Cnrip1*	443.2455	58.0123	-2.9337	0.00950304
ENSMUSG00000034422	*Parp14*	106.3987	14.6432	-2.8612	0.000215992
ENSMUSG00000017830	*Dhx58*	443.2993	61.8703	-2.8410	2.15E-05
ENSMUSG00000040483	*Xaf1*	2073.6151	305.2619	-2.7640	3.11E-06
ENSMUSG00000028125	*Abca4*	507.2879	74.9403	-2.7590	2.65E-08
ENSMUSG00000030413	*Pglyrp1*	1769.1796	266.4742	-2.7310	2.58E-05
ENSMUSG00000009350	*Mpo*	153024.9743	23725.1902	-2.6893	2.31E-10
ENSMUSG00000073902	*Gm1966*	356.1311	56.7955	-2.6486	3.48E-05
ENSMUSG00000052730	*Gm5111*	553.8990	88.6299	-2.6438	0.00605291
ENSMUSG00000018930	*Ccl4*	591.6837	95.1808	-2.6361	0.042286013
ENSMUSG00000051839	*Gypa*	322.0535	52.1228	-2.6273	0.04342323
ENSMUSG00000046456	*Tmem150b*	1297.7010	212.7118	-2.6090	0.000238521
ENSMUSG00000033191	*Tie1*	60.5123	10.0979	-2.5832	0.025645645
ENSMUSG00000039831	*Arhgap29*	145.3590	24.8101	-2.5506	0.007754229
ENSMUSG00000003206	*Ebi3*	1433.9443	245.2095	-2.5479	6.46E-05
ENSMUSG00000037578	*Pkd2l1*	572.1308	99.2655	-2.5270	1.84E-05
ENSMUSG00000031239	*Itm2a*	3110.9241	550.6514	-2.4981	1.29E-06

ENSMUSG00000042284	*Itga1*	1244.3144	222.5806	-2.4830	8.71E-07
ENSMUSG00000079018	*Ly6c1*	974.6151	174.8242	-2.4789	0.004712114
ENSMUSG00000021751	*Acox2*	159.5378	29.7298	-2.4239	0.020479066
ENSMUSG00000075602	*Ly6a*	45367.3408	8462.5709	-2.4225	2.12E-08
ENSMUSG00000062545	*Tlr12*	298.2285	55.7606	-2.4191	0.000296739
ENSMUSG00000024910	*Ctsw*	624.2473	117.1062	-2.4143	0.000584082
ENSMUSG00000000782	*Tcf7*	1407.0269	269.6332	-2.3836	2.99E-05
ENSMUSG00000042671	*Rgs8*	915.1571	177.9536	-2.3625	0.004960299
ENSMUSG00000021262	*Evl*	2568.6465	515.5648	-2.3168	2.56E-06
ENSMUSG00000038418	*Egr1*	2495.0490	515.6167	-2.2747	1.72E-06
ENSMUSG00000031712	*Il15*	698.2529	152.5926	-2.1941	0.016613297
ENSMUSG00000050377	*Il31ra*	161.6534	35.5191	-2.1862	0.020875159
ENSMUSG00000015316	*Slamf1*	292.9910	64.7548	-2.1778	0.023379706
ENSMUSG00000029605	*Oas1b*	1065.1100	238.3209	-2.1600	0.002129662
ENSMUSG00000050232	*Cxcr3*	435.0837	98.1933	-2.1476	0.006041361
ENSMUSG00000078612	*1700024P16Rik*	299.0975	68.1296	-2.1343	0.00295208
ENSMUSG00000078853	*Igtp*	5703.5639	1327.5082	-2.1031	1.52E-05
ENSMUSG00000023132	*Gzma*	23052.7543	5374.3681	-2.1008	8.69E-07
ENSMUSG00000053541	*Gm4759*	656.9892	153.9629	-2.0933	0.002277311
ENSMUSG00000051314	*Ffar2*	3384.0613	809.3256	-2.0640	1.75E-05
ENSMUSG00000053168	*9030619P08Rik*	4753.3753	1140.9747	-2.0587	0.002511814
ENSMUSG00000026222	*Sp100*	1439.2239	356.5118	-2.0133	0.000161424
ENSMUSG00000030107	*Usp18*	4955.7992	1237.3509	-2.0019	1.04E-05
ENSMUSG00000027368	*Dusp2*	11771.9232	2942.1792	-2.0004	3.85E-06
